# Baroreflex sensitivity impairment in Long-COVID patients: a diagnostic tool for classifying the autonomic dysfunction spectrum

**DOI:** 10.3389/fcvm.2026.1830347

**Published:** 2026-07-14

**Authors:** Alejandro Sáinz-Jiménez, Ignacio Romero Fragoso, Guadalupe Estrella Salazar Calderon, Santiago Martinez-Falcon, Hannah Molinari Luna, Jesus Portocarrero Nieto, Andrea Barajas-Aguilar, Antonio Barajas-Martínez, Isael Guillermo García Macedo, Felipe Gonzalez-Alvarez, Maria Silvia Lopez-Yañez, Brayans Becerra-Luna, Raul Martinez-Memije, Erwin Chiquete, Carlos Cantu, Karla Maria Tamez-Torres, Jose Sifuentes-Osornio, Claudia Lerma, Ruben Fossion, Tania Reyes-Cruz, Bruno Estañol, Jose de Jesus Aceves-Buendia

**Affiliations:** 1 Facultad de Ingeniería, Universidad Nacional Autónoma de México, Ciudad de México, México; 2 Departamento de Neurología y Psiquiatría, Instituto Nacional de Ciencias Médicas y Nutrición Salvador Zubirán, Ciudad de México, México; 3 Unidad de investigación de enfermedades metabólicas INCMNSZ, Tecnológico de Monterrey, Ciudad de México, México; 4 Departamento de Fisiología Cardíaca, Instituto Nacional de Cardiología Ignacio Chávez, Ciudad de México, México

**Keywords:** baroreflex, BRS, dysautonomia, long-covid, orthostatic-intolerance, POTS

## Abstract

**Background:**

Long-COVID describes a variety of COVID-19 side effects lasting longer than three months. Among these side effects are cardiovascular alterations, such as Postural Orthostatic Tachycardia Syndrome (POTS), caused by an autonomic nervous system dysfunction. A higher incidence of POTS and decreased baroreceptor sensitivity (BRS) has been reported in Long-COVID patients. Many of these patients present orthostatic intolerance similar to that observed in POTS, which does not strictly coincide with the criteria that have previously been established for POTS subtypes. Therefore, we aim to determine if the decrease of the baroreceptor sensitivity is enough to diagnose different degrees of the autonomic dysfunction spectrum.

**Methods:**

A cross-sectional study was conducted in a cohort of individuals who presented with various long-term symptoms for at least four weeks after a moderate acute COVID-19 infection. To further evaluate orthostatic intolerance (OI), we developed a new method that enables a more detailed characterization of cardiovascular dynamics using beat-to-beat physiological time series. Since these dynamics can be assessed through the measurement of baroreceptor sensitivity, this new method employs a geometric analysis that reveals varying degrees of baroreceptor sensitivity impairment. The proposed method generated a graph of baroreflex sensitivity that consistently showed a decrease in this index.

**Results:**

Patients exhibited significantly lower BRS compared with healthy controls during orthostatism. Furthermore, we noticed that patients with lower BRS had a significantly higher arterial blood pressure and heart rate, as well as an overall lower heart rate variability. The proposed method also correlated with previously recognized canonical variables of HRV, as well as being validated with the sequence method. Additionally, this allows us to understand and reclassify patients’ diagnoses within the spectrum of symptoms similar to postural orthostatic tachycardia syndrome (POTS).

**Conclusions:**

The proposed method allowed us to consider this decrease in baroreflex sensitivity measurement as a diagnostic tool through a spectrum-based approach to reclassify patients. This analysis can be incorporated into the set of variables considered to improve the diagnosis of patients with Long-COVID.

## 1 Introduction

Following the SARS-CoV-2 pandemic, patients who survive an acute COVID-19 infection present with a wide variety of symptoms and signs 3–6 months after infection. This set of symptoms is commonly referred to as Long-COVID (1, 2).

Among these sequelae, dysautonomia and orthostatic intolerance have been identified. Alongside several cases of Postural Tachycardia Syndrome (POTS), its incidence has significantly increased from 1.42 to 20.3 cases per 1,000,000 person-years (3); specifically, between 9% and 61% of post-infected people have reported symptoms similar to those of POTS patients, e.g., POTS-like symptoms (4–7).

POTS-like symptoms have been previously reported following other acute viral infections (8, 9), and it has been hypothesized that they may be associated with an abnormal inflammatory response (10–12) specifically in the brainstem (13, 14).

These symptoms are referred to as Orthostatic Intolerance (OI), a broad term encompassing a group of heterogeneous pathologies with similar clinical features, including changes in Heart Rate (HR) and arterial Blood Pressure (BP) upon standing (15–18).

Some of the symptoms associated with OI are syncope, blood pooling in the lower limbs, and an altered baroreflex sensitivity with a change in the sympathetic nervous response; this leads to several symptoms, such as dizziness, blurry vision, fatigue, and palpitations. All these symptoms appear or worsen upon standing and are relieved or diminished by returning to the supine position (15).

Among the pathologies described in OI are POTS, orthostatic hypotension, vasovagal syncope, immediate orthostatic hypotension, and cardioinhibitory syncope, among others (15).

POTS is a type of OI characterized by an excessive rise in HR equal to or greater than 30 bpm (or a rate that exceeds 120 bpm) with little or no change in blood pressure during the first 10 min of standing. Currently, the recognized POTS subtypes are as follows: hyperadrenergic, hypovolemic, and neuropathic (19–21).

Among these subtypes, the hyperadrenergic subtype is characterized by excessive tachycardia and an increase in Systolic Blood Pressure (SBP) ≥ 10 mmHg during the first 10 min of standing, accompanied by elevated levels of norepinephrine (>600 ng/mL) (18). These features suggest that sympathetic hyperactivity is particularly common in this subgroup of POTS patients (22, 23).

Particularly, survivors of a mild COVID-19 infection showed a decrease in baroreflex sensitivity (BRS), defined as a response in HR frequency to the BP changes to maintain homeostasis (24) and an increase in carotid artery stiffness (25); this was also shown after severe COVID-19, although improvement was reported after 27 weeks (26).

These BRS changes have been documented in Long-COVID patients with different degrees of POTS severity during the active orthostatism test (27) as well as in the supine position (28).

Traditional methods for measuring BRS require longer measurements as well as complex algorithms and procedures in both the time domain and the frequency domain (e.g., sequence method and cross-spectral analysis) (29–32). These methods are often difficult to replicate consistently (e.g., Valsalva maneuver) or require specialized devices or pharmacological interventions (e.g., Neck chamber, Oxford method) (33–35).

In this regard, with the ever-rising Long-COVID trajectories and its different sequelae (36), POTS-like symptoms and their severity need to be re-evaluated to establish a classification range for patients who do not meet the criteria for hyperadrenergic POTS. Therefore, the following exploratory study aims to propose a classification of patients with mild COVID-19, based on the change in baroreflex sensitivity, which could serve as a diagnostic tool for understanding dysautonomia and POTS-like symptoms.

The present exploratory study aims to determine whether a multivariable analysis and Heart Rate variability (HRV) can be used to phenotypically differentiate and more precisely characterize subtypes of mild hyperadrenergic POTS. Compared with traditional methods, our BRS graphical method aims to be easier to replicate and evaluate.

## 2 Methods

This study was approved by the Research Ethics Committee at the National Institute of Medical Sciences and Nutrition Salvador Zubirán, which gave ethical approval for this work with reference number INF-4452 23-24-1.

### 2.1 Study population

Patients were selected from a limited database of individuals who had been infected with mild to moderate COVID-19, meaning that none of the patients were previously hospitalized and presented symptoms at least four weeks after the acute state of infection (1). The pathological group is majoritarily composed of women 82%, in line with previous Long-COVID findings (37).

Patients were initially screened using the COMPASS 31 questionnaire, alongside a modified version of the same questionnaire, COMPASS 31+, as shown in Online Resource 1, designed to screen for COVID-19 sequelae (*n* = 38). Subsequently, a control group, without comorbidities such as diabetes, high blood pressure, or other conditions, was selected (*n* = 14) (Online Resource 2). Although an *a priori* sample size calculation was not performed due to the limited availability of eligible patients and the exploratory nature of the study, a *post hoc* power analysis was later conducted based on the observed effect sizes, and it was determined that the estimated sample size required to achieve 90% statistical power would range approximately from 3 to 13 subjects per group, depending on the comparison. The selected power levels were based on conventional criteria for sample size estimation and study design, as described by Cohen's conventional criteria (38).

Once these patients were selected, they underwent an active orthostatism test, during which their BP and HR were measured using a Finapress Finometer with a finger sensor (39), continuously monitoring and collecting hemodynamic data beat-by-beat sampling at 200 Hz.

The following protocol was applied to all patients: the device was placed on the ring finger of the left hand; they were then asked to lie immobile, breathe normally, without sighs, and remain silent in the supine position for seven minutes. After this period, patients were asked to stand up unassisted and remain standing immobile for seven minutes while breathing normally. Finally, they were subsequently asked to return to the supine position.

### 2.2 Steady state analysis

Once the SBP, HR, and inter-beat interval (IBI) were obtained, statistical analysis was performed.

All patient data were analyzed following the same procedure. First, we selected the data periods of 5 min corresponding to the steady states of SBP, HR, and IBI values during the supine and orthostatic positions; steady states were defined as the periods 1 min after the change in position when the SBP and HR had mostly settled (no statistical difference in mean and variance). These stable periods were established when the patients were not moving and there were no external perturbations, when applicable, artifacts were manually filtered and removed based on anomalous amplitude and discontinuity of surrounding data. Once this was done, we calculated the delta (Δ) value between the mean measurement of each beat position for the SBP, IBI, and HR. Additionally, the means and standard deviations were calculated for the previously mentioned variables.

To quantify the HRV, we used the Kubios Lite software version 4.2.0 to calculate the linear variables: The percentage of successive NN (R-R) heartbeat intervals that differ by more than 50 milliseconds, (pNN50), Standard deviation of the NN (R-R) intervals (SDNN), and Root mean square of successive differences (RMSSD), and also to calculate the non-linear *α*2 from the Detrended Fluctuation Analysis (DFA) for each steady state. Furthermore, to evaluate autonomic dysfunction in the frequency domain, the Low frequency (LF) band, the High frequency (HF) band, and the LF/HF ratio were analyzed in normalized units (n.u.). These values were calculated using the same 5 min window (40) as the selected steady states with a linear detrend, using no additional filters or beat correction, an acceptance threshold of 5%, with an interpolation rate of 4 Hz and the “Smoothn priors” interpolation method and a 500 smoothing parameter.

In addition, we used a Poincaré graph with a 95% confidence level for the IBI to observe the beat-by-beat regularity and dispersion, as well as to calculate the Standard deviation of Poincaré plot perpendicular to the line-of-identity (Sd1) and the standard deviation of the Poincaré plot along the line-of-identity (Sd2) values to measure eccentricity for the adjusted orbit.

### 2.3 BRS analysis

To measure baroreflex sensitivity, we developed an innovative graphical methodology (41) based on the ΔIBI and ΔSBP, as described by Swenne et al. (42) and Gouveia et al. (43), who define BRS as the change in inter-beat interval in milliseconds per unit of change in blood pressure in mmHg.

To calculate the BRS using this graphical method, we used data corresponding to the previously selected SBP and IBI steady states. Once selected, the differences between successive points were calculated to obtain values representing the pressure and HR changes per beat (ΔSBP and ΔIBI, respectively).

Subsequently, we placed both Delta (Δ) values on a scatter plot with ΔSBP on the *X* axis and ΔIBI on the *Y* axis; then, an ellipse was adjusted over the data with 95% confidence, utilizing chi square in order to have a proper characterization of the interaction between the variables with enough certainty.

Following the adjustment, we extracted the ellipse equation in its canonical form: $Ax^{2}+Bxy+Cy^{2}+Dx+Ey+F=0$. In order to properly analyze this data, a reescalation of one of the axis was needed since ΔIBI is expressed in milliseconds and ΔSBP in mmHg; the numerical range of ΔIBI is approximately one order of magnitude larger than ΔSBP. This anisotropic scaling affects the apparent orientation of the fitted ellipse. To avoid the effects of this distortion, we introduced a fixed normalization factor k = 10 corresponding to the ratio (ΔIBI/ΔSBP) between the characteristic scales of the two axes. Given that the set of points used to fit our ellipse are as follows: (ΔSBP, ΔIBI), then x = ΔSBP and y = ΔIBI we define the normalize coordinate $y*=\;\frac{y}{k}$, then y=ky* into the ellipse equation gives as a result:

$$Ax^{2}+kBxy*+k^{2}Cy{*}^{2}+Dx+kEy*+F=0$$

And given the previously mentioned formula to get the inclination angle of the ellipse is only dependant on A, B, and C the coefficients transform as follows:

$$A*=A\;B*=kB\;C*=k^{2}C$$

Resulting in the following values:

A*=AB*=10BC*=100C

**Figure 1:**
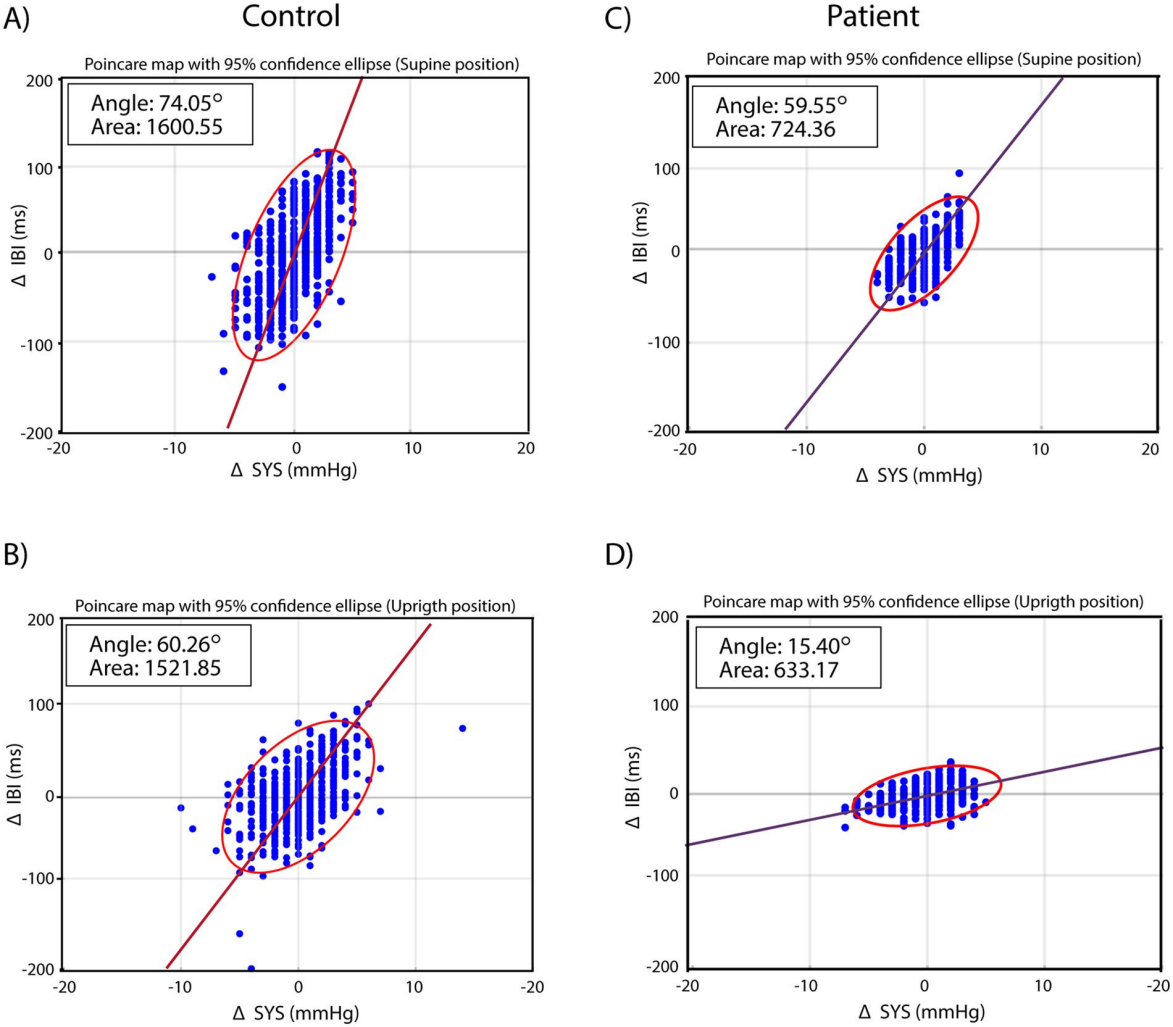
Graphical BRS shown by theta angle. The Theta graphics corresponding to the supine **(A,C)** and orthostatic **(B,D)** positions of a healthy subject are shown. We show the scatter plot of the ΔSBP and ΔIBI values in blue, over which a 95% confidence ellipse was adjusted and is shown in red; additionally, a dark red line with the calculated Theta [*θ*] angle was overlaid. The axis scales were normalized to observe the natural response without altering the raw delta (Δ) values. **(A)** Theta graph during the supine period for a control subject. **(B)** Theta graph during the orthostatism period for a control subject. **(C)** Theta graph during the supine period for a pathological subject. **(D)** Theta graph during the orthostatism period for a pathological subject.

These scaling factors are the direct consequence of rescaling the ΔIBI axis to match the order of magnitude of ΔSBP before calculating the ellipse rotation angle. Once this normalized factor was applied, we calculated the ellipse rotation angle against the *X* axis, using a modified version of the standard rotation axis formula for conic equations to obtain what we refer to as the patient's “Theta Angle” [θ] (Figure 1).

$$\cot(2\theta )\;=\frac{A-C}{B}$$

From where we cleared the *θ* variable to obtain:

$$\theta =\frac{\mathrm{co}t^{-1}\left(\frac{A-C}{B}\right)}{2}$$

It is important to mention that this normalization does not modify the raw beat-to-beat ΔSBP and ΔIBI time series; it only changes the coordinate system used to estimate the ellipse orientation. This is consistent with the figure description indicating that the axis scales were normalized without altering the raw delta values; this was performed for all patients, ensuring reproducibility.

Additionally, to the abovementioned method, a simplified analog method was developed alongside it in which we normalize the data of the ΔSBP and ΔIBI by dividing each value by 10 and 100, respectively. Once this was done, we fit an ellipse with 95% confidence, and we proceed to calculate the semi-major axis of the ellipse so that we may obtain the rotation angle with respect to the horizontal axis by calculating the inverse tangent of the slope formed by the previously mentioned semi-axis to obtain the patient's Theta Angle [θ]. It's important to note that this method requires the raw data to be re-escalated as well as the area of the ellipse.

**Figure 2:**
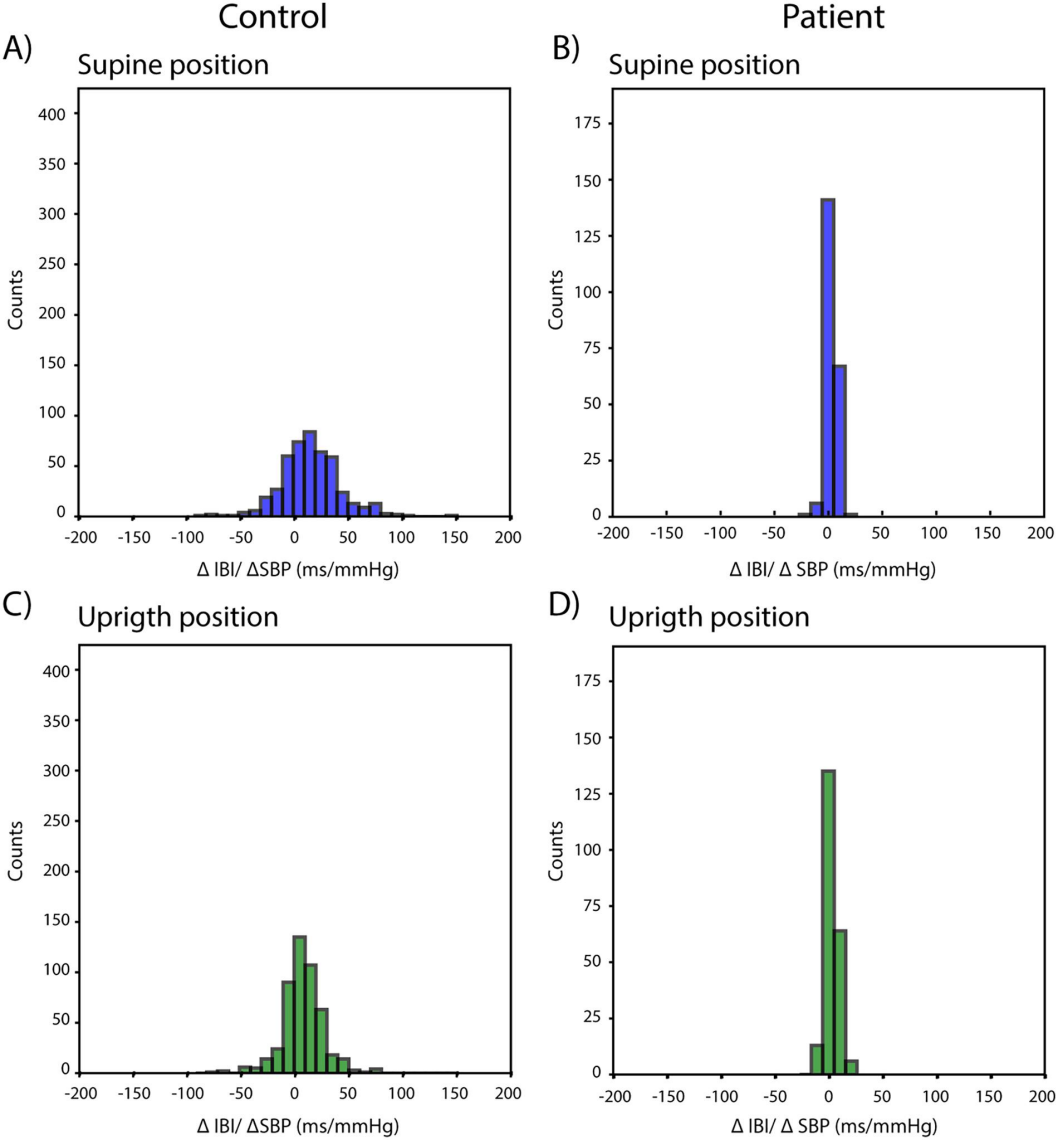
Distribution of standard deviation of BRS values. Histogram showing the BRS values corresponding to the supine **(A,B)** and orthostatic **(C,D)** positions, obtained from the division of ΔIBI/ΔSYS beat-to-beat measurements. Distribution of the BRS values for a control subject during the supine **(A)** and upright positions **(C)** Distribution of the BRS values for a pathological subject during the supine **(B)** and upright **(D)** positions.

Furthermore, we calculated the BRS using the difference between successive IBI values divided by the difference between successive SBP values, for which we also calculated their dispersion values, such as the mean, standard deviation (SD), and kurtosis (Figure 2). Likewise, we validated this proposed BRS measurements with the sequence method as the gold standard. Based on the resulting data from this analysis, we consider that we could also segregate the pathological groups within mild hyperadrenergic POTS.

The results are reported as mea*n* ± SD; statistical significance was assessed using the Mann–Whitney test, as well as a multiple comparisons correction with the Bonferroni test. A two-tailed *p*-value of at least <0.05 was considered statistically significant.

### 2.4 Diagnostic performance analysis

We evaluated the diagnostic performance of the canonical and newly proposed variables for predicting OI positivity due to Long-COVID, using the COMPASS 31 test, enriched with complementary questions related to autonomic symptoms in Long-COVID patients (COMPASS 31+), which served as the gold standard. The diagnostic performance was assessed by calculating the sensitivity, specificity, positive predictive value (PPV), and negative predictive value (NPV).

Accuracy was estimated by the area under the receiver operating characteristic (ROC) curve for continuous variables. For categorical variables, accuracy was calculated as follows: accuracy = (true positives + true negatives)/total number of persons. We also calculated the Youden index (J) for efficiency and selection of cut-off values, when relevant.

## 3 Results

We performed the COMPASS 31 + test on all patients and found an increase in symptoms associated with the sympathetic system activity (COMPASS 31 + score: 45.55 ± 24.94) and with COMPASS 31 score >20 (44).

### 3.1 Long-COVID patients show heterogeneous responses during an active orthostatism test

**Figure 3:**
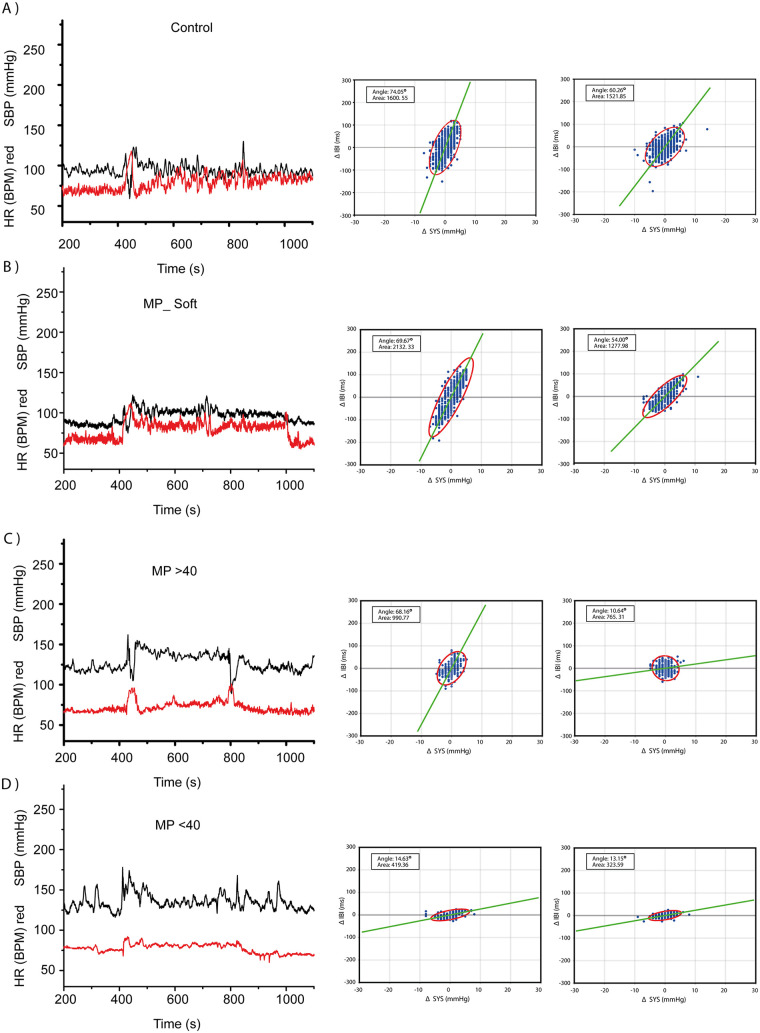
Different degrees of BRS impairment. Measured SBP and HR values (colored in black and red, respectively) of one patient from each of our selected groups, along with the BRS response represented by the Theta angle for the supine and upright positions. **(A)** SBP and HR values of a healthy control accompanied by their respective Theta graphs. **(B)** SBP and HR values accompanied by their respective Theta graphs of a patient with Mild POTS and a soft change in the BRS response. **(C)** SBP and HR values accompanied by their respective Theta graphs of a patient with Mild POTS and a change in angle >40° for the BRS response. **(D)** SBP and HR values accompanied by their respective Theta graphs of a patient with Mild POTS and an attenuated BRS response.

BP and HR changes in two positions (supine and orthostatism) were evaluated using Finapress. None of the patients met the criteria for hyperadrenergic POTS. Nevertheless, all exhibited orthostatic increases in both HR and SBP. Therefore, there is clearly a wide distribution of BP and HR delta (Δ) values (13.72 ± 12.66 mmHg, *p* = 0.005; 13.62 ± 6.67 bpm, *p* = 0.746), with corresponding coefficient of variation values (SBP: 0.923, HR: 0.489). Thus, patients did not constitute a homogeneous group (Figure 3).

### 3.2 BRS determined subgroups

**Figure 4:**
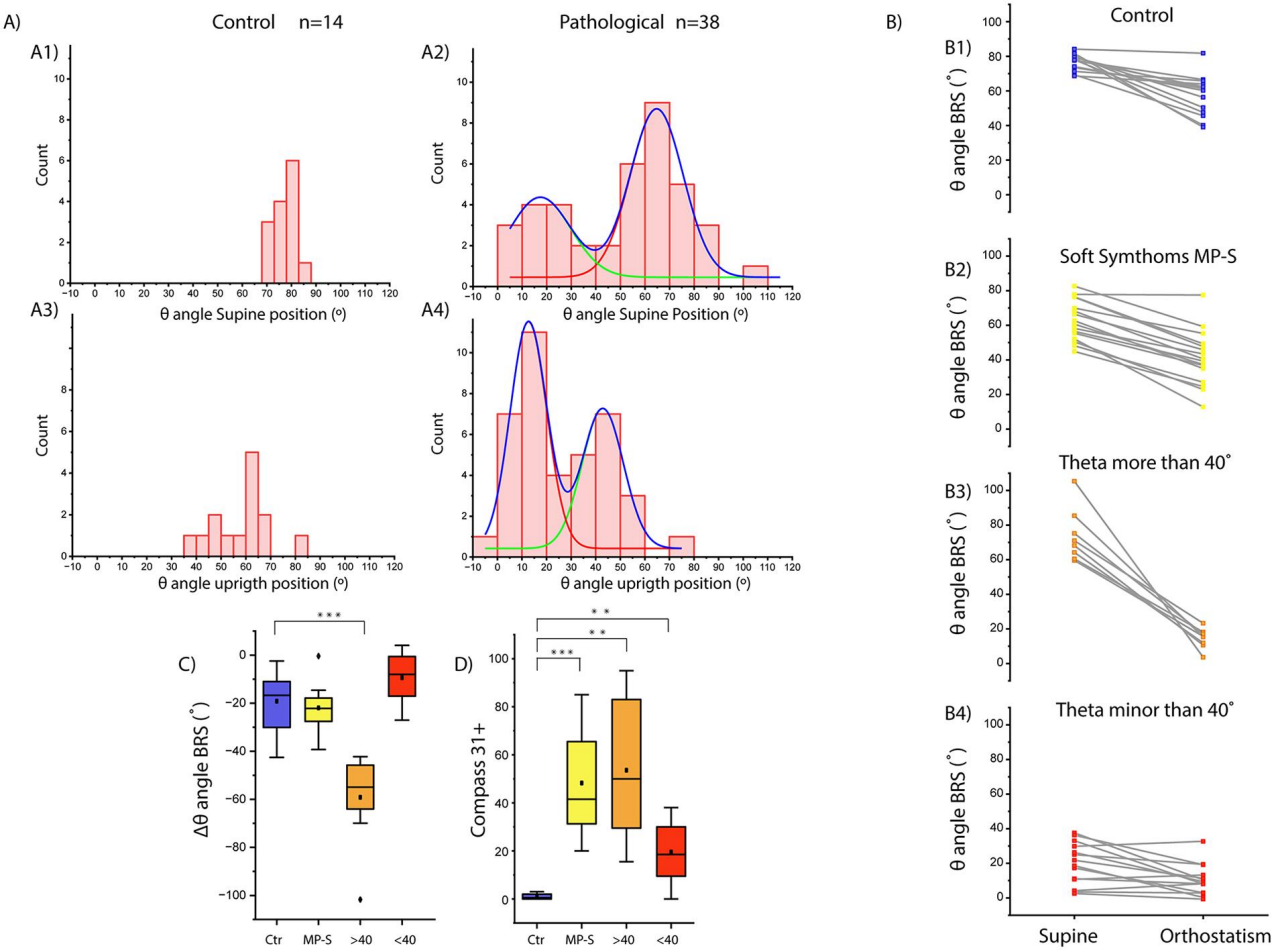
Theta angle shows a bimodal distribution that separates the distinct groups of mild POTS. **(A)** Histogram of the control and patient groups for the Theta value during supine and upright positions. **(A1)** Histogram of the supine Theta measurements for the control group; the scale was adjusted to match that of the patient group. **(A2)** Histogram of the supine Theta measurements for all patients; two Gaussian curves were adjusted above the graph, since it was observed that the data showed bimodality. **(A3)** Histogram of the upright Theta measurement for the control group; the scale was adjusted to match that of the patient group. **(A4)** Histogram of the upright Theta measurements for all patients; two Gaussian curves were adjusted above the graph, since it was observed that the data showed bimodality. **(B)** We have placed the Theta [*θ*] values for the supine (left) and orthostatism (right) periods of all study subjects; these values were connected by a line to their corresponding value, and each group was assigned a color (blue for controls, yellow for MP-S, orange for MP Δ*θ* > 40 and red for the MP *θ* < 40 group). **(B1)** Theta values for the supine and upright positions of the control group. **(B2)** Theta values for the supine and upright position of the MP-S group. **(B3)** Theta values for the supine and upright position of the MP Δ*θ* > 40 group. **(B4)** Theta values for the supine and upright position of the MP *θ* < 40 group. **(C)** Box plot for the ΔTheta [*θ*] value of all groups, where each group was assigned a color (blue for controls, yellow for MP-S, orange for MP Δ*θ* > 40 and red for the MP *θ* < 40 group). **(D)** Box plot for the COMPASS 31 + score of the patients in each of the selected groups. To indicate statistical significance to the control group, an asterisk [*] was placed for a *p* < 0.05, two asterisks [**] for *p* < 0.001 and three asterisks [***] for *p* < 0.0001.

Consequently, we decided to measure the BRS with the graphical method for the Theta angle in both the supine and orthostatic positions. We observed a bimodal distribution of Theta supine angle (θs: 51.09 ± 26.43, coefficient of variation: 0.51747) in all patient populations during the supine period (Figure 4C).

Based on our patient sample the Theta supine (θs) values seemed to fit a bimodal distribution; the two populations intersected at a supine Theta value of 40.651° this value allows us to set an initial and exploratory classification for the present study. Then, we decided to perform a subtraction between Theta ortho (θo) and Theta supine (θs); this is a delta Theta (Δθ=θo−θs) for an additional classification feature (Figure 4).

Thus, we determined that the pathological groups could be classified into 3 distinct groups based on this division of the two populations θs and the third group based on the following: 1) Mild POTS with a Δθ change equal to or greater than 40° (MP 40° *n* = 8); 2) Mild POTS with a Theta supine value (θs) that starts below 40° and remains so during the upright position (MP *θ* < 40 = *θ*s ≤ 40° & *θ*o ≤ 40° *n* = 14); and 3) Mild POTS with less OI symptoms that does not meet the above criteria; this has the softest change in Δθ (MP-S *n* = 16) (Figure 4).

**Figure 5:**
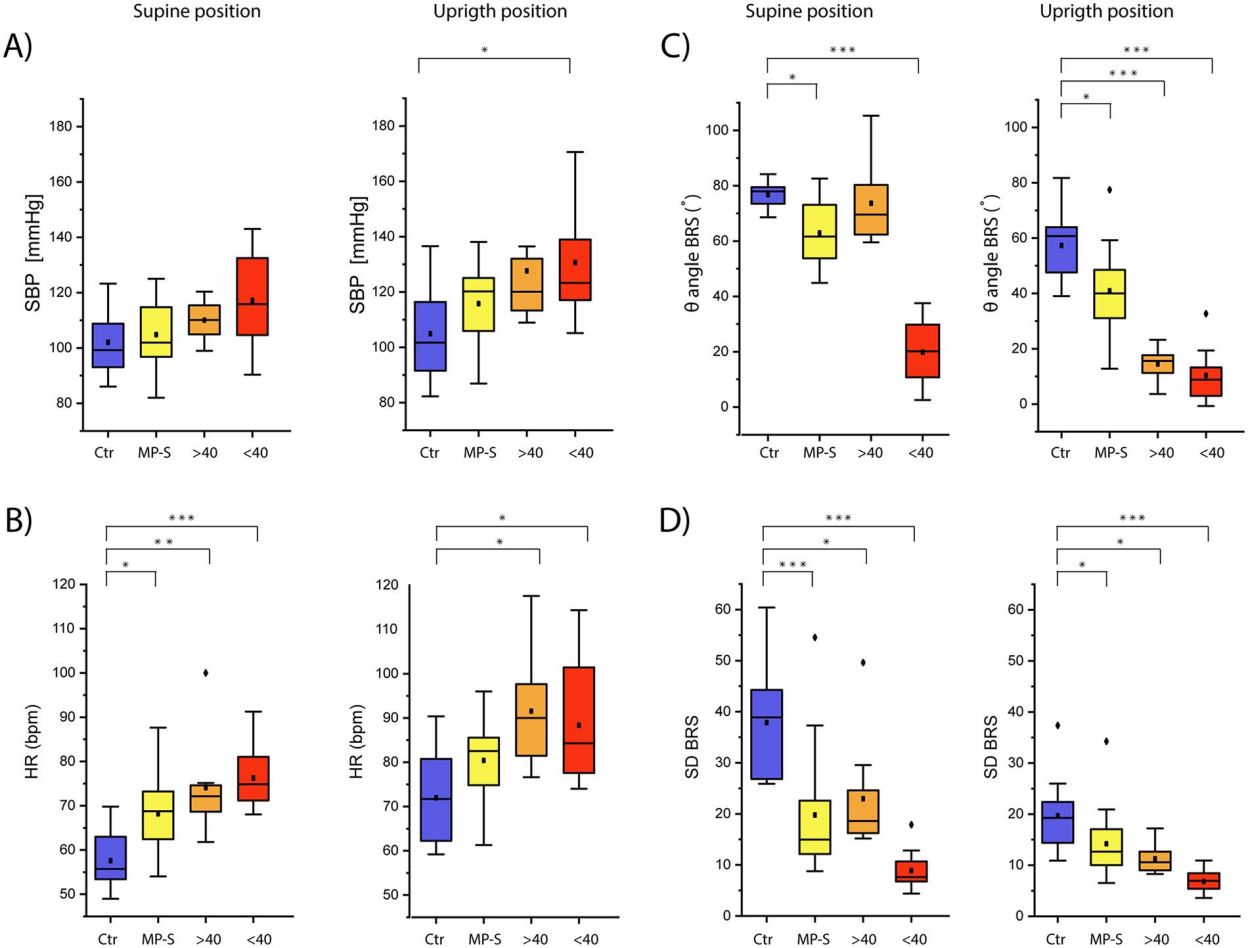
The decrement of BRS increases HR and SBP. Box Plots for SBP **(A)**, HR **(B)**, Theta **(C)**, and the SD of the BRS histogram **(D)** during the supine (left) and upright position (right). To indicate statistical significance to the control group, an asterisk [*] was placed for a *p* < 0.05, two asterisks [**] for *p* < 0.001 and three asterisks [***] for *p* < 0.0001.

During the supine period, the control group did not show significant differences in SBP when compared with POTS subgroups, during orthostatism, the SBP of the control group (104.85 ± 16.30) was significantly different from the MP *θ* < 40 group (130.59 ± 20.10; *p* = 0.024) (Figure 5A).

Regarding the HR control during the supine position, significant differences were observed (control: 57.56 ± 6.72) compared to MP-S (68.17 ± 8.55; *p* = 0.004), MP Δ*θ* > 40 (74.08 ± 11.27; *p* = 0.001) and MP *θ* < 40 (76.25 ± 6.55; *p* < 0.001), as well as during orthostatism for the MP Δ*θ* > 40 and MP *θ* < 40 (control 71.96 ± 9.86 vs. MP Δ*θ* > 40 91.55 ± 13.22, *p* = 0.001; and vs. MP *θ* < 40 88.36 ± 12.64, *p* = 0.001) (Figure 5B).

The delta values of HR between supine and orthostatic positions (ΔHR) did not differ significantly between the control and the POTS subgroups. In contrast, the ΔSBP value was significantly different for the MP Δ*θ* > 40 (11.11 ± 12.72; *p* = 0.029) groups compared to that of the control group (2.89 ± 9.58) (Table 1).

**Table 1:** Shows the mean and standard deviation for all variables across all groups, significant *p*-values performed with the Bonferroni test have been highlighted in bold and with an asterisk (*).

Variable	Control		MP-S		p	MP Δ*θ* > 40		*p*	MP θ < 40		p
	Mean	SD	Mean	SD	Control-MP-S	Mean	SD	Control-MP Δθ > 40	Mean	SD	Control-MP θ < 40
SBP Sup	102.019	11.153	104.788	13.213	1.000	110.030	7.419	1.000	117.154	16.977	0.089
SBP Ortho	104.858	16.307	115.780	13.940	0.763	127.616	23.459	0.061	130.594	20.101	**0**.024*
Δ SBP	2.895	9.584	13.053	6.717	0.159	18.778	21.016	**0**.029*	11.117	12.727	0.476
HR Sup	57.565	6.729	68.172	8.553	**0**.004*	74.081	11.280	**0**.001*	76.252	6.557	**<0**.0001*
HR Ortho	71.963	9.861	80.401	9.545	0.260	91.550	13.223	**0**.001*	88.367	12.646	**0**.001*
Δ Hr	14.050	5.344	12.254	4.974	1.000	17.880	5.858	1.000	12.194	8.094	1.000
Escent. IBI Sup	0.652	0.094	0.609	0.172	1.000	0.579	0.235	1.000	0.829	0.056	**0**.012*
Escent. IBI Ortho	0.806	0.075	0.799	0.083	1.000	0.850	0.035	0.968	0.832	0.059	1.000
Sd2/Sd1 Sup	1.831	0.357	1.859	0.641	1.000	1.711	0.447	1.000	3.648	1.031	**<0**.0001*
Sd2/Sd1 Ortho	3.126	0.849	3.222	1.209	1.000	3.586	0.667	1.000	3.481	0.991	1.000
θ Sup	76.713	4.663	62.905	11.536	**0**.007*	73.660	15.325	1.000	19.811	12.110	**<0**.0001*
θ Ortho	57.319	11.681	40.924	15.635	**0**.002*	14.486	5.851	**<0**.0001*	10.375	8.842	**<0**.0001*
Δ θ	−19.215	12.523	−21.981	8.480	1.000	−59.174	19.379	**<0**.0001*	−9.436	10.451	0.236
θ Area Sup	3,485.474	1,384.141	1,663.354	1,236.926	**0**.001*	1,998.458	2,005.416	0.068	760.432	413.310	**<0**.0001*
θ Area Ortho	2,224.996	1,109.874	1,211.592	504.566	**0**.013*	1,738.400	1,281.812	1.000	862.373	538.420	**0**.001*
BRS Mean Sup	18.443	4.570	12.548	9.757	0.084	7.159	4.886	**0**.001*	2.976	2.041	**<0**.0001*
BRS Mean Ortho	7.439	3.411	5.796	2.674	0.511	2.010	1.881	**<**.0001*	1.946	1.531	**<0**.0001*
BRS SD Sup	37.877	10.547	19.776	12.160	**<0**.0001*	22.959	11.675	**0**.008*	8.872	3.567	**<0**.0001*
BRS SD Ortho	19.673	6.855	14.183	6.641	**0**.045*	11.241	2.917	**0**.005*	6.814	2.112	**<0**.0001*
BRS Kurt Sup	5.447	4.879	3.407	2.824	1.000	3.857	3.537	1.000	6.888	11.576	1.000
BRS Kurt Ortho	4.490	3.718	5.007	5.754	1.000	5.811	5.661	1.000	9.825	19.300	1.000
LF/HF Sup	0.892	0.536	1.590	3.892	1.000	0.990	1.061	1.000	3.342	2.706	0.122
LF/HF Ortho	2.032	1.422	1.732	1.560	1.000	2.134	1.949	1.000	2.595	2.074	1.000
SDNN Sup	65.607	18.163	39.075	18.854	**0**.001*	37.113	12.398	**0**.001*	20.286	7.904	**<0**.0001*
SDNN Ortho	53.364	17.184	36.025	16.341	**0**.028*	32.325	13.004	**0**.028*	20.543	15.960	**<0**.0001*
RMSSD Sup	77.357	20.334	45.856	25.260	**0**.001*	46.013	21.250	**0**.004*	16.886	5.134	**<0**.0001*
RMSSD Ortho	50.971	23.904	33.556	22.670	0.248	28.663	16.890	0.188	21.464	24.182	**0**.007*
pNN50 Sup	47.939	12.417	24.071	24.011	**0**.001*	15.775	13.237	**0**.001*	0.819	1.613	**<0**.0001*
pNN50 Ortho	25.346	18.903	7.404	8.724	**0**.001*	5.553	8.242	**0**.001*	0.967	1.328	**<0**.0001*

Poincaré plots were generated from the IBI values of all patients to evaluate the dispersion of continuous beat-to-beat data; after that, eccentricity was measured for the supine and orthostatic Poincaré graphs (Supplementary Figure S1). It was observed that the MP *θ* < 40 group had a higher eccentricity value during the supine period (0.82 ± 0.05), which was significantly different than that of the following groups: control (0.65 ± 0.09; *p* = 0.012), MP-S (0.60 ± 0.17; *p* < 0.001), and MP Δ*θ* > 40 (0.57 ± 0.23; *p* = 0.001). However, during the orthostatic period, there were no significant differences between the groups.

We also measured the relationship between the Sd2 and Sd1 of the Poincaré graph of the IBI alongside the eccentricity of the ellipse and found that the MP *θ* < 40 was significantly different from those of all other groups (Supplementary Figure S1).

### 3.3 BRS decrease in long-COVID patients

For the BRS measured by the Theta (*θ*) values during the supine period, the MP-S (62.90 ± 11.53) and MP *θ* < 40 (19.81 ± 12.11) groups were significantly different from that of the control group (76.71 ± 4.66), with *p* = 0.007 for MP-S and *p* < 0.001 for the MP *θ* < 40 group, while the MP Δ*θ* > 40 group was not. On the other hand, during the orthostatic period, the mean values of all the groups differed from those of the control group (57.31 ± 11.68), MP-S (40.92 ± 15.63; *p* = 0.002), MP Δ*θ* > 40 (14.48 ± 5.85; *p* < 0.001), and MP *θ* < 40 (10.37 ± 8.84; *p* < 0.001) (Figure 5C).

The mean BRS values were measured by the ΔIBI/ΔSBP [ms/mmHg] coefficient by group; these values also show a decrease between the groups: 18.44 ± 4.56 for the control group, 12.54 ± 9.75 for the MP-S, 7.15 ± 4.88 for the MP Δ*θ* > 40, and 2.97 ± 2.04 for the MP *θ* < 40 in the supine position; and 7.43 ± 3.41 for the control group, 5.79 ± 2.67 for the MP-S, 2.01 ± 1.88 for the MP Δ*θ* > 40, and 1.94 ± 1.53 for the MP *θ* < 40 in the upright position (Table 1).

These differences are validated by the pre-existing sequence method and by the relationship between our new Theta variable and the measured positive and negative sequences of the BRS; the Spearman's R value is  > 0.7 for all values. This correlation validates the use of our own method (Supplementary Figure S2) and was also performed for the SD BRS measurement (Supplementary Figures S3, S4).

#### 3.3.1 BRS dispersion measures

The mean of the BRS (ΔSBP/ΔIBI) was only significantly lower in the MP Δ*θ* > 40 and MP *θ* < 40 in the supine period (control: 18.44 ± 4.56; MP-S: 12.54 ± 9.75, *p* = 0.084; MP Δ*θ* > 40: 7.15 ± 4.88, *p* = 0.001; MP *θ* < 40: 2.97 ± 2.04, *p* < 0.001); in the upright position, MP Δ*θ* > 40 (2.01 ± 1.88) and MP *θ* < 40 (1.94 ± 1.53) groups were significantly different from the healthy control group (7.43 ± 3.41), with *p* < 0.001 for both comparisons (Table 1).

The standard deviation of the BRS was also studied, as it was observed in the individual histograms that patients exhibited reduced variability compared to healthy individuals. In this regard, the standard deviation for all patient groups was significantly different from that of the control group during the supine period (control: 37.87 ± 10.54; MP-S: 19.77 ± 12.16, *p* < 0.001; MP Δ*θ* > 40: 22.95 ± 11.67, *p* = 0.008; MP *θ* < 40: 8.87 ± 3.56, *p* < 0.001); this is also true for the upright position,(control: 19.67 ± 6.85; MP-S: 14.18 ± 6.64, *p* = 0.045; MP Δ*θ* > 40: 11.24 ± 2.91; *p* = 0.005; MP *θ* < 40: 6.81 ± 2.11; *p* < 0.001); (Figure 5D).

### 3.4 POTS subgroups are supported by canonical HRV variables

**Figure 6:**
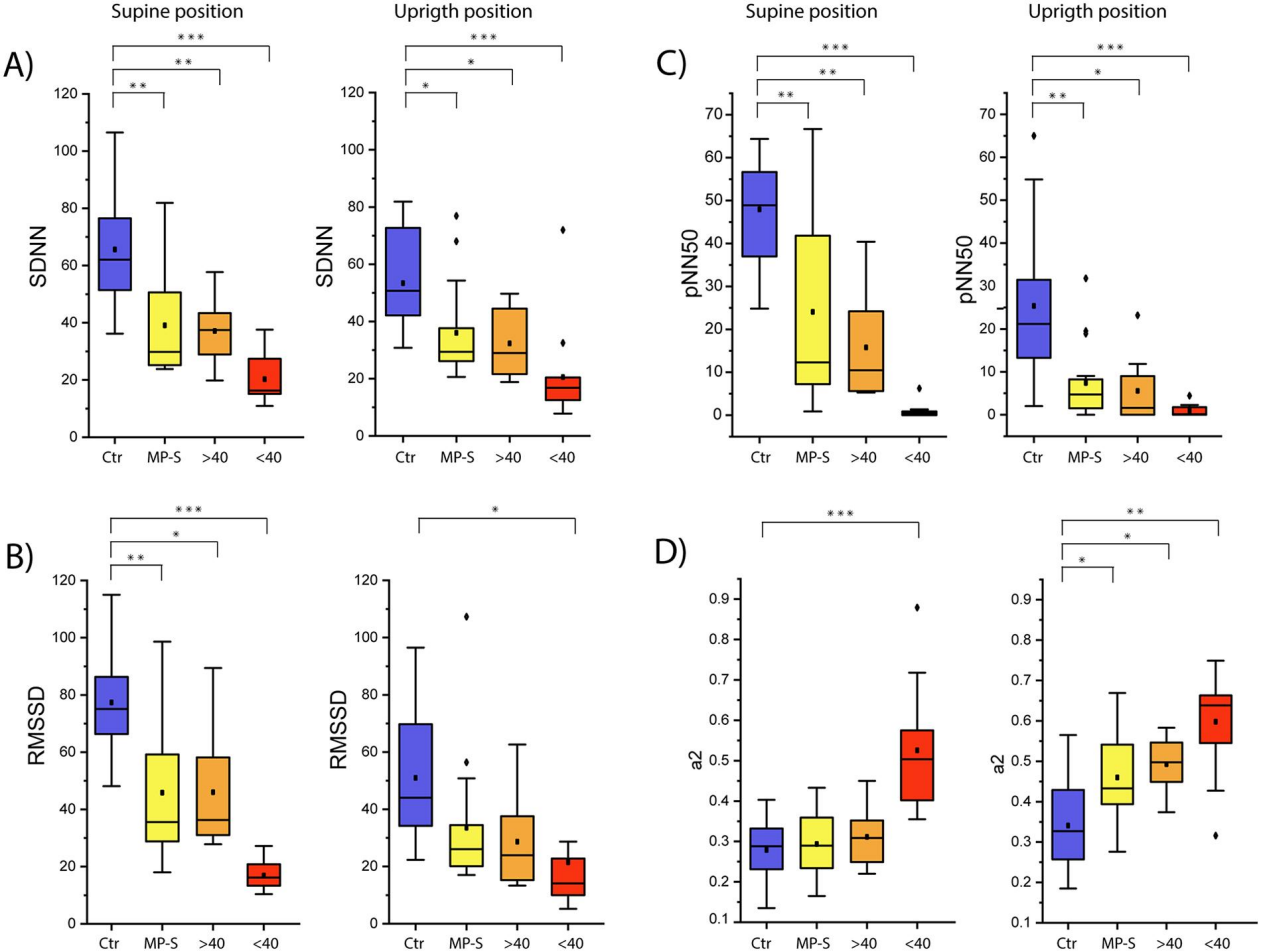
Canonical variables decrease in the different groups of mild POTS delimited by theta angle. Box plots of the SDNN **(A)**, RMSSD **(B)**, pNN50 **(C)** and a2 **(D)** for each of the groups in the supine (left) and upright (right) position. To indicate statistical significance to the control group, an asterisk [*] was placed for a *p* < 0.05, two asterisks [**] for *p* < 0.001 and three asterisks [***] for *p* < 0.0001.

In the supine position, all patient groups had significantly different values for the SDNN (Figure 6A left) (control: 65.60 ± 18.16; MP-S: 39.07 ± 18.85, *p* < 0.001; MP Δ*θ* > 40: 37.11 ± 12.39, *p* < 0.001; MP *θ* < 40: 20.28 ± 7.90, *p* < 0.001), RMSSD (Figure 6B left) (control: 77.35 ± 20.33; MP-S: 45.85 ± 25.26, *p* < 0.001; MP Δ*θ* > 40: 46.01 ± 21.25, *p* = 0.004; MP *θ* < 40: 16.88 ± 5.13, *p* < 0.001) and pNN50 (Figure 6C left) (control: 47.93 ± 12.41; MP-S: 24.07 ± 24.01, *p* < 0.001; MP Δ*θ* > 40: 15.77 ± 13.23, *p* < 0.001; MP *θ* < 40: 0.81 ± 1.61, *p* < 0.001). In addition, the MP *θ* < 40 group had SDNN values below 50 ms (45), RMSSD values lower than 30 ms (46), and pNN50 values lower than 3% (47); all of them are considered high-risk values.

The values were also significantly different during the orthostatic period for the SDNN (Figure 6A right) (control: 53.36 ± 17.18; MP-S: 36.02 ± 16.34, *p* = 0.028; MP Δ*θ* > 40: 32.32 ± 13.00, *p* = 0.028; MP *θ* < 40: 20.54 ± 15.95, *p* < 0.001), and pNN50 (Figure 6C right) (control: 25.34 ± 18.90; MP-S: 7.40 ± 8.72, *p* < 0.001; MP Δ*θ* > 40: 5.5525 ± 8.24167, *p* = 0.001; MP *θ* < 40: 0.96 ± 1.32, *p* < 0.001). For the RMSSD (Figure 6B right) only the MP *θ* < 40 (21.46 ± 24.18, *p* = 0.007) was significantly different from the control (control: 50.97 ± 23.90).

The Theta angle in the MP *θ* < 40 group was defined by low variability with a pNN50 range of 0–2.29 (ms), an RMSSD range of 5.2–45.64 (ms) and a collapsed ellipse area of 285.82–1,400.79. Compared with the control, the MP-S group presented a lower pNN50; however, the MP *θ* < 40 group presented the lowest values compared to the others.

### 3.5 Canonical variables correlate with new variables

#### 3.5.1 HRV variables vs. BRS theta

HRV variables are known to decrease in POTS and Long-COVID patients (27, 48–50), however, the correlation between these variables and the BRS in the POTS groups remains unclear. Therefore, scatter plots were generated with the HRV variables on the *X*-axis and the proposed variables (Theta and BRS SD) on the *Y*-axis.

To characterize the behavior of the data, we attempted to fit mathematical functions to describe the relationship between the two sets of variables. Based on the observed pattern, an S-shaped logistic function was fitted to the supine graph data. Since the S-shaped function did not fit the orthostatic period graph data properly, we implemented a logistic function.

**Figure 7:**
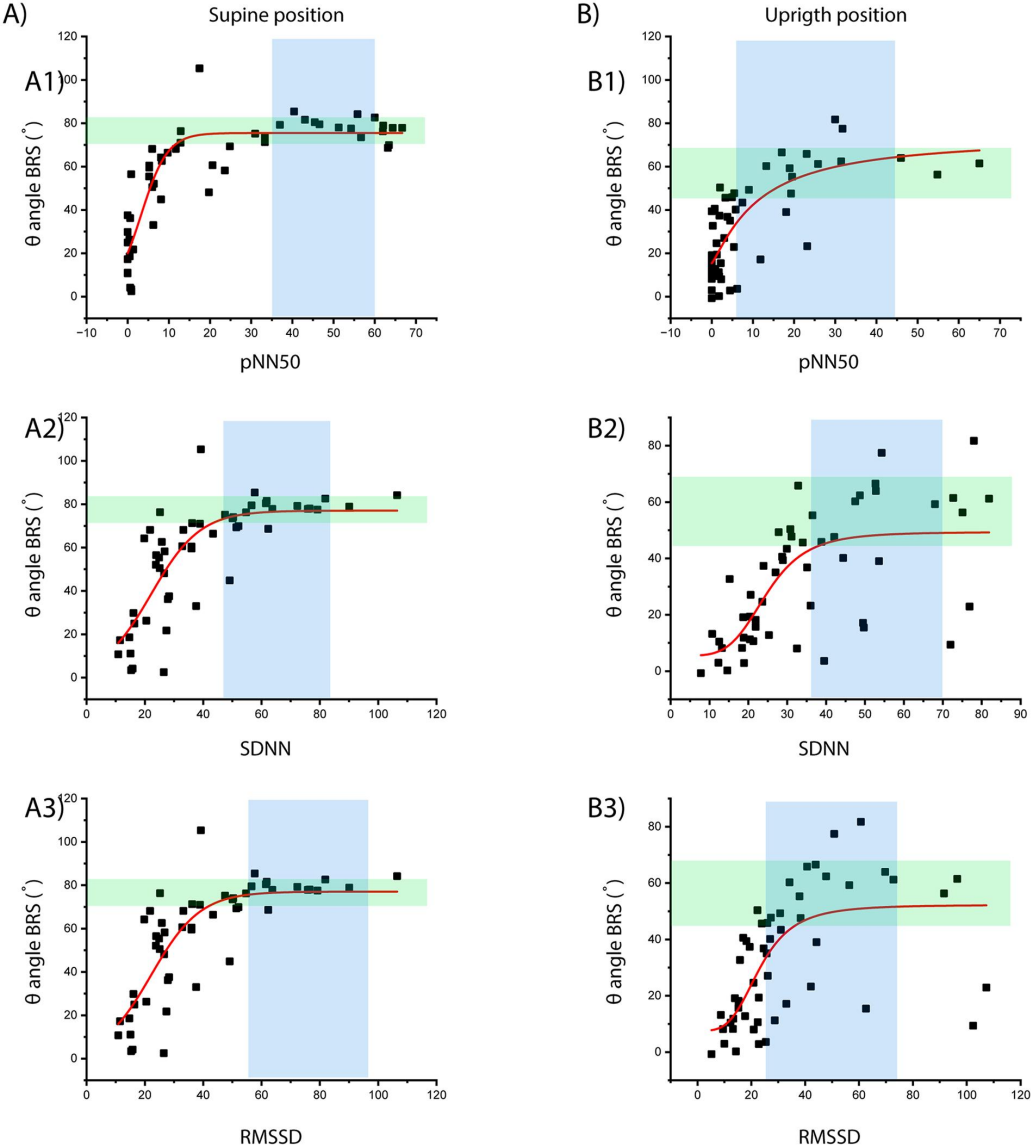
Relationship between canonical HRV variables and theta angle of BRS. HRV variables have been placed on the *X*-axis to compare them with the Theta values, which have been placed on the *Y*-axis in a scatter plot. Additionally, the range of the control group has been highlighted with a blue and green rectangle. **(A1)** Scatter plot of the pNN50 and Theta values during the supine position with an adjusted S-Logistic function. **(A2)** Scatter plot of the SDNN and Theta values during the supine position with an adjusted S-Logistic function. **(A3)** Scatter plot of the RMSSD and Theta values during the supine position with an adjusted S-Logistic function. **(B1)** Scatter plot of the pNN50 and Theta values during the upright position with an adjusted Logistic function. **(B2)** Scatter plot of the SDNN and Theta values during the upright position with an adjusted Logistic function. **(B3)** Scatter plot of the RMSSD and Theta values during the upright position with an adjusted Logistic function.

The Theta value was strongly correlated with all canonical variables. The strongest correlations were observed between the Theta value and the supine RMSSD (Spearman's rho = 0.9) and pNN50 (Spearman's rho = 0.85), suggesting that our Theta value has a significant influence on parasympathetic measurements. Additionally, the relationship between the SDNN (Spearman's rho = 0.83, Spearman test) and the Theta value is very strong, leading us to conclude that BRS-Theta can be associated with an individual's HRV autonomic response (Figure 7).

#### 3.5.2 HRV variables vs. SD BRS

**Figure 8:**
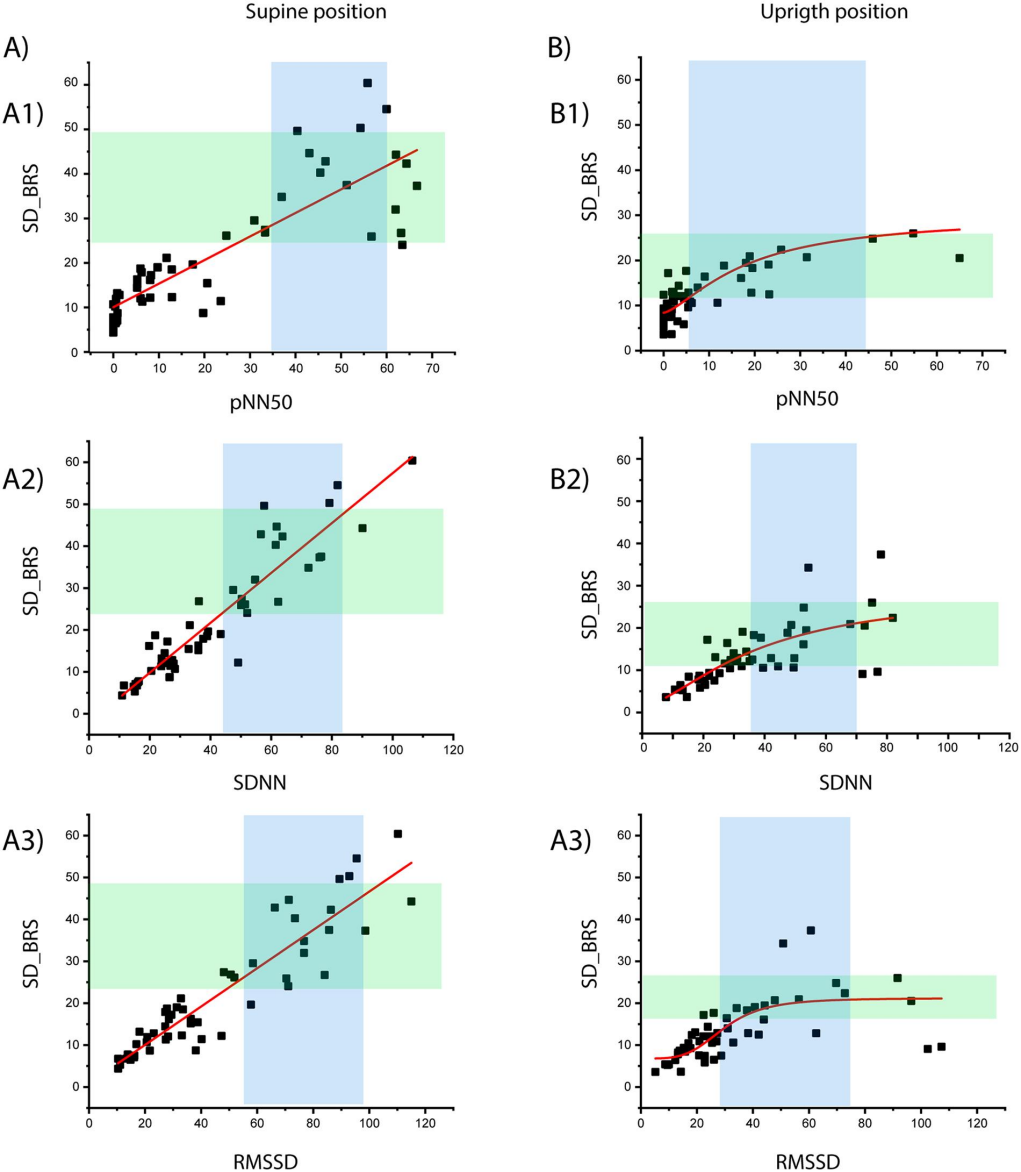
Standard deviation of the BRS with respect to the HRV canonical variables. HRV variables have been placed on the *X*-axis to compare them with the SD BRS values obtained, which have been placed on the *Y*-axis in a scatter plot. Additionally, the range of the control group has been highlighted with a blue and green rectangle. **(A1)** Scatter plot of the pNN50 and SD BRS values during the supine position with an adjusted S-Logistic function. **(A2)** Scatter plot of the SDNN and SD BRS values during the supine position with an adjusted linear function. **(A3)** Scatter plot of the RMSSD and SD BRS values during the supine position with an adjusted linear function. **(B1)** Scatter plot of the pNN50 and SD BRS values during the upright position with an adjusted Logistic function. **(B2)** Scatter plot of the SDNN and SD BRS values during the upright position with an adjusted linear function. **(B3)** Scatter plot of the RMSSD and SD BRS values during the upright position with an adjusted Logistic function.

To validate and describe the data behavior from the proposed variables, we performed a correlation between the SD of the BRS and the HRV variables; this relationship was shown using a linear regression during the supine position for all variables. During the upright position, a logistic function was applied (Figure 8).

**Figure 9:**
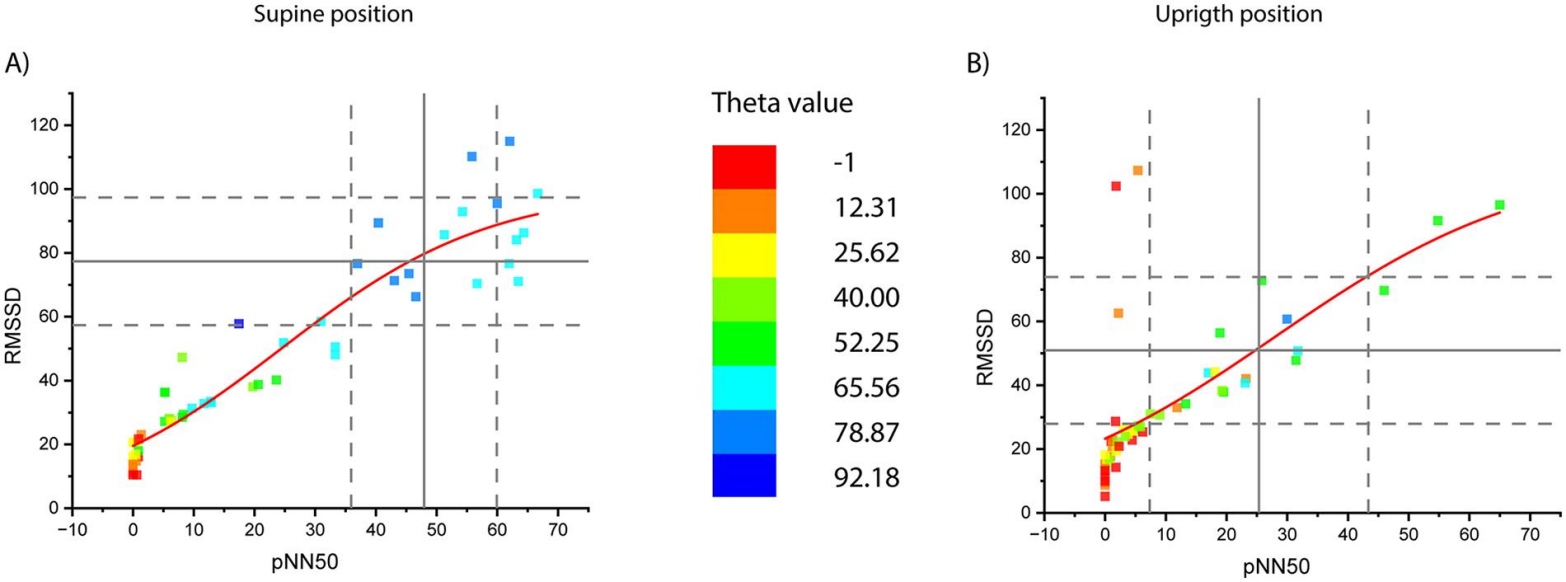
Progression of BRS impairment. Scatter plot of RMSSD as a function of pNN50 in the supine position **(A)** and orthostatism **(B)** The corresponding theta values are also shown (color gradient).

To evaluate how the patient groups correlated with the HRV values, three axes graphs were generated corresponding with our variables and the HRV variables to describe the progression of BRS dysfunction (Figure 9).

It is important to note that the intersection of the two standard deviations of the controls on the two axes was close to the Theta value of 40°, as mentioned above (green color); this value divides the subgroups.

**Figure 10:**
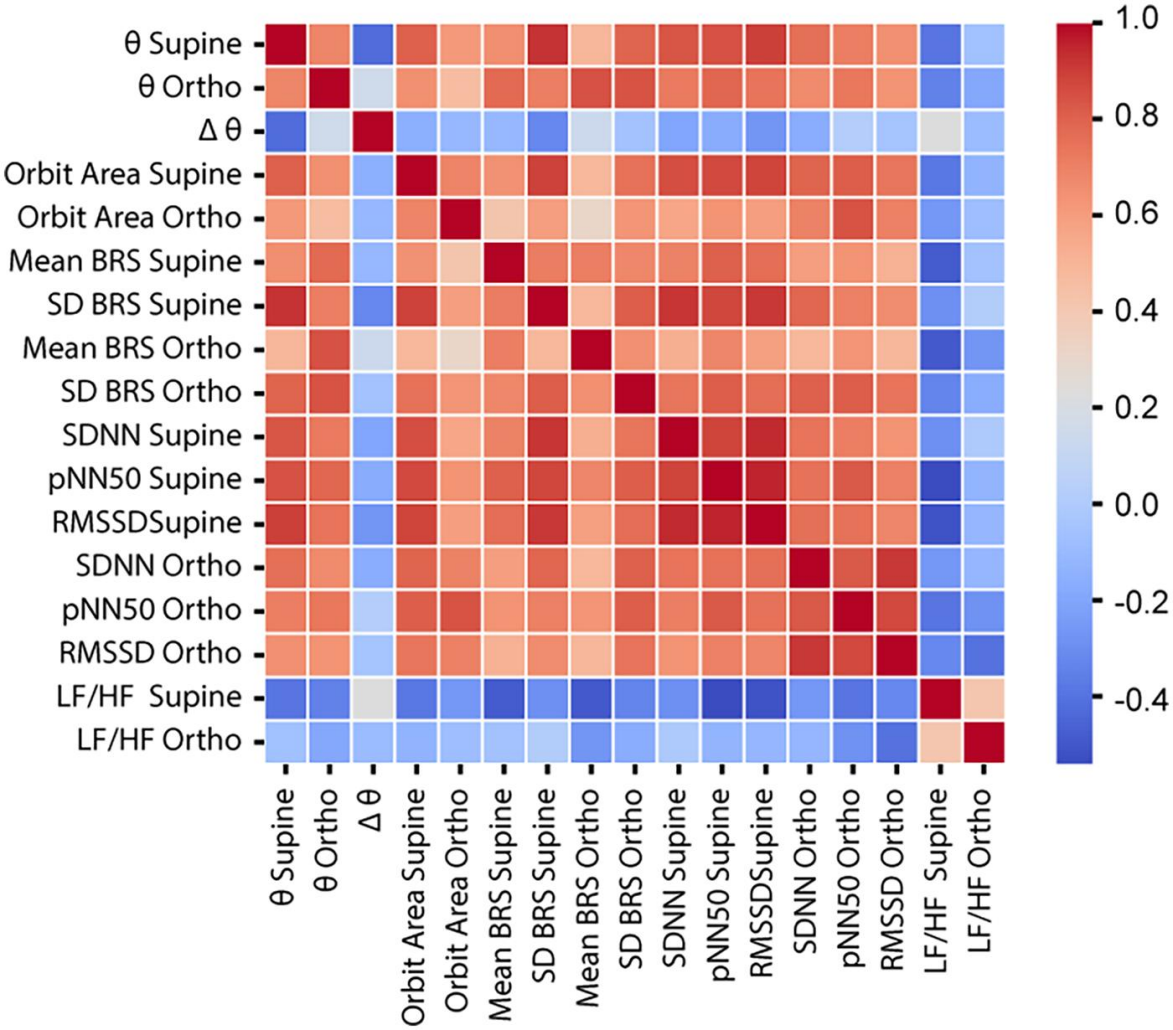
Spearman significance between canonical and new variables. Matrix with the canonical HRV and proposed Theta and SD BRS values during the supine and upright positions, where a heatmap was applied to match the correlation values obtained using the Spearman’s test.

Furthermore, a Spearman's correlation test was performed on the newly proposed variables and the canonical HRV variables, yielding a very strong correlation; most of the correlations were positive and exceeded a rho value of 0.5, with the correlation between the supine Theta and the supine SD BRS being the highest, with a rho value of 0.92. Spearman's test was used because the correlations did not show a linear relationship (Figure 10).

**Figure 11:**
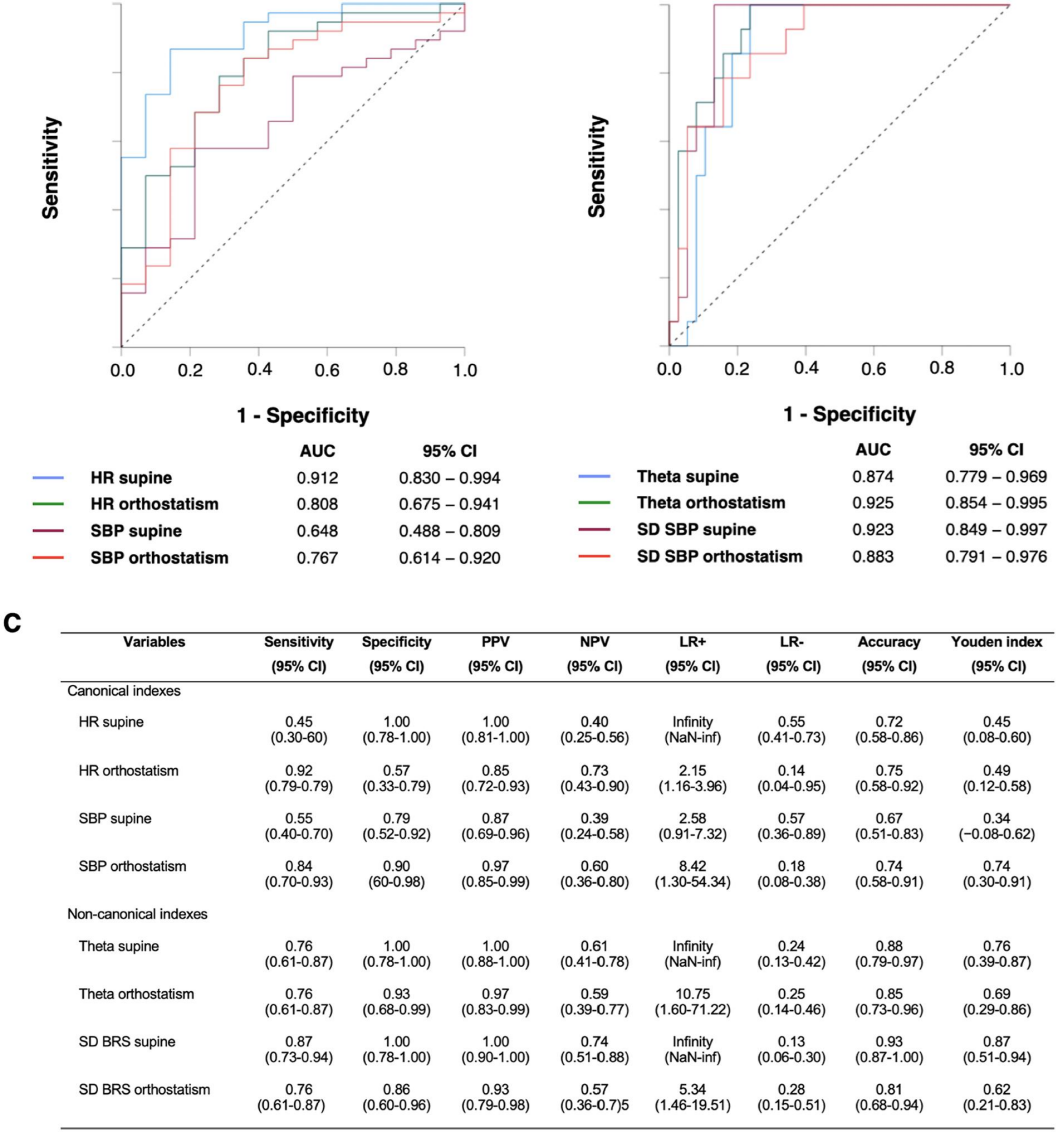
ROC curves for SBP, HR, and proposed variables. Receiver operating characteristics (ROC) curve showing the cut-off quest and accuracy analyses for canonical **(A)** and non-canonical **(B)** finometry Mild POTS indexes with the composite autonomic symptom score COMPASS 31 as the gold standard. Table **(C)** showing the results of the diagnostic performance for several cut-off values found by ROC analyses for the sets of canonical and non-canonical indexes.

In addition to the previously described analysis, ROC curves were generated to demonstrate the specificity and sensitivity of the proposed variables. Along with these new variables, SBP, HR and HRV were also measured. This analysis revealed that canonical variables are useful but not sufficient compared with our proposed variables, which are more accurate (>0.81). However, together, they can be used to establish a pathological diagnosis (Figure 11). Additionally, the following cut-off points were established for the proposed variables: Theta supine: 68.39 [°]; Theta Ortho: 38 [°]; SD BRS supine: 24.97; and SD BRS orthostatism: 12.84.

In summary, when examining the percentage change in BRS relative to the control group, we observed decreases of 17.99% in the MP-S group, 3.97% in the MP Δ*θ* > 40 group, and 74.17% in the MP *θ* < 40 group for the supine position. During the orthostatic position, the percentage decrease in the control group was 25.28%, while for the patient groups it reached 46.65% for the MP-S group, 81.11% for the MP Δ*θ* > 40 group, and 86.47% for the MP *θ* < 40 group.

In conclusion, a decrease in the BRS function was observed in all our groups; specifically, the decrease in the BRS function was more pronounced during the orthostatic position. Furthermore, the MP Δ*θ* > 40 has the greatest change, corresponding to patients with the most severe symptoms.

## 4 Discussion

In the present exploratory study, we compared asymptomatic control patients with a cohort of patients experiencing Long-COVID, who presented a heterogeneous range of symptoms.

Using our analytical approach, we found that: (1) patients with Long-COVID frequently exhibited higher Heart Rate (HR), together with a reduced baroreflex sensitivity (BRS) response, compared with controls; (2) patients with Long COVID showed lower Heart Rate variability (HRV) values; and (3) decreased BRS was correlated with reduced HRV. Taken together, these findings suggest the presence of varying degrees of autonomic dysfunction, which may be interpreted as a physiological spectrum.

Based on these observations, we propose that a decrease in BRS can be considered as a possible diagnostic tool for classifying patients who present POTS-like symptoms in Long-COVID.

It has been reported that patients with Long-COVID may exhibit autonomic dysfunction characterized by sympathetic hyperactivity and impaired cardiovagal function (51, 52). This frequently manifests clinically as orthostatic intolerance (OI) (27), which is consistent with the physiological alterations observed in our cohort.

Previous studies have explored the sequelae of Long-COVID using different approaches to assess autonomic function through Heart Rate variability (HRV) analysis, aiming to better understand the pathophysiology of orthostatic intolerance (OI).

In this context, Ormiston et al. (7) and Hira et al. (27) evaluated orthostatic intolerance (OI) and compared these findings with patients diagnosed with POTS, reporting that survivors of acute COVID-19 infection frequently exhibited features consistent with POTS and autonomic dysfunction.

Similarly, Azcue et al. (28) compared Long-COVID patients presenting orthostatic intolerance (OI) with patients diagnosed with myalgic encephalomyelitis/chronic fatigue syndrome (ME/CFS) and reported an increase in HR, further supporting the presence of autonomic imbalance.

Additionally, Keller et al. (51) compared the autonomic function of patients with Long COVID and patients with pure autonomic failure, reporting that individuals with Long COVID exhibited autonomic dysfunction patterns similar to those observed in pure autonomic failure.

Importantly, their study also described the presence of an indeterminate or nondiagnostic subgroup that was excluded from the final classification. This subgroup may still present autonomic dysfunction, although without a sufficiently high probability of overt clinical autonomic failure. These observations raise the possibility that subtypes of POTS-like dysautonomia may exist that are not clearly identifiable using current diagnostic criteria.

Likewise autonomic dysfunction is associated with BRS impairment specifically, Srivastava et al. (25) and Zanoli et al. (26) identified that mild and severe COVID-19 patients had decreased BRS during the supine position, this is also true for the study made by La Rovere et al. (53).

However, in these studies, patients were not classified according to the severity of BRS dysfunction. Consequently, given that BRS dysfunction of COVID-19 patients does not manifest homogeneously, our observations support the interpretation of this alteration as a physiological spectrum. Based on this, we propose that patients may be classified into the following subgroups: MP-S, MP Δ*θ* > 40, and MP*θ* < 40.

The proposed classification was validated using the sequence method (54, 55). Using this independent approach, the four groups followed a pattern of progressive BRS impairment similar to that observed with the *θ* angle, further supporting that patients with Long COVID exhibit an attenuated baroreflex response.

Importantly, this agreement between two distinct analytical methods (sequence method and graphical method) supports that the observed subgrouping is not merely a consequence of the *θ*-based classification framework itself, but rather reflects an underlying physiological structure present in the data.

Notably, the *θ* angle provided improved segregation of the pathological subgroups, whereas the pre-established sequence method more broadly classified the control group across a wider physiological spectrum (Supplementary Figure S2). Nevertheless, both methods consistently corroborated the existence of the proposed groups (Supplementary Figure S5), providing additional support for the robustness and non-circularity of our findings.

Consistent with these observations, our comparison between Long COVID patients presenting orthostatic intolerance and healthy controls showed a reduction in both baroreflex sensitivity (BRS), assessed through the proposed graphical method, and Heart Rate variability (HRV), further supporting the presence of autonomic dysfunction.

Previous frequency-domain HRV studies in Long COVID such as Santos-de-Araujo et al. (52) and Marques et al. (56) reported an increase in the low-frequency (LF) band together with a decrease in the high-frequency (HF) band, resulting in an altered LF/HF ratio. This pattern is widely interpreted as indicative of autonomic imbalance, characterized by increased sympathetic activity and reduced parasympathetic activity.

On the other hand, González-Hermosillo et al. (57) compared patients with Long-COVID and healthy controls and reported similar responses between groups in the frequency domain, in the LF/HF ratio, suggesting no clear evidence of persistent autonomic dysfunction after a prolonged recovery period following infection.

However in our study, we identified changes in the LF/HF ratio, using the Mann Whitney test (*p* = 0.001), between controls and specific pathological subgroups (Supplementary Figure S6), which is consistent with the findings reported by Santos-de-Araujo et al. (52).

These findings suggest that subgroup-specific autonomic alterations may be overlooked when Long-COVID is analyzed as a single homogeneous entity and only analyzing HRV, further supporting the relevance of our proposed physiological stratification based on BRS.

In this regard autonomic dysfunction is closely linked to baroreflex sensitivity (BRS), as a reduction in BRS is frequently accompanied by decreased Heart Rate variability (HRV), as has been described in conditions such as decompensated chronic heart failure and hypertension (58–60).

Under these circumstances, the high-frequency (HF) band typically decreases, reflecting impaired parasympathetic modulation. In addition, some patients exhibit an increase in the low-frequency (LF) band, resulting in an altered LF/HF ratio (52, 56, 61), which has been associated with varying degrees of autonomic dysfunction (51).

Our results are consistent with these observations, further supporting that the physiological alterations identified in our Long COVID subgroups are compatible with different levels of autonomic imbalance.

Other pathologies also exhibit varying degrees of BRS impairment. For example, diabetic patients show a decrease of BRS. Healthy individuals show a proportional change in the IBI measurement for every 1 unit of SBP change; this is referred to as a change of ∽5–10 ms per mmHg (46, 62). In contrast, diabetic patients have previously shown a decrease in BRS of approximately 30%–50% (63–66) due to two factors: 1) a decrease in HRV and 2) autonomic dysfunction caused by structural damage to the vagal afferent and brainstem control. Notably, these alterations can be demonstrated even before a fully diagnosed diabetic state (46, 67). Thus, these patients may be classified with mild or severe BRS dysfunction (68).

Similarly to the mild BRS dysfunction described in diabetic patients, our cohort also exhibited varying degrees of BRS reduction, which further supports the proposal of a spectrum-based approach to autonomic dysfunction in Long-COVID.

On the other hand, several viral infections have been associated with brainstem dysfunction and the development of POTS-like symptoms (69, 70). Similarly, SARS-CoV-2 infection has been reported to affect brainstem structures and to induce a neuroinflammatory response (71, 72).

This neuroinflammation may alter the firing rates of local brainstem nuclei as well as baroreceptor adaptation mechanisms, thereby impairing central autonomic regulation and potentially contributing to the development of POTS-like symptoms (13, 73, 74).

A similar phenomenon has also been observed in animal models, in which lipopolysaccharide-induced systemic inflammation leads to a reduction in baroreflex sensitivity (BRS) (75), further supporting a mechanistic link between inflammation and autonomic dysfunction.

In our patients, the BRS decrease was approximately 80% in the group with the most severe symptoms; this means less adaptability to postural changes, resulting in impaired cardiac vagal baroreflex and autonomic nervous system dysfunction. This has strong clinical implications since less autonomic adaptability could manifest as fluctuating hypertension or presyncope symptoms (76, 77).

Based on these observations, we propose a novel graphical method for estimating baroreflex sensitivity (BRS) that may facilitate rapid physiological assessment without necessarily requiring complex algorithms or specialized software for interpretation.

This approach directly generates a graphical representation that can be immediately interpreted through the theta angle [*θ*] and the corresponding ellipse area, thereby allowing an intuitive visualization of the interaction between systolic blood pressure (SBP) and Heart Rate (HR) dynamics.

As demonstrated by the Spearman correlation analysis, these newly derived variables were also significantly associated with changes in Heart Rate variability (HRV), further supporting their physiological relevance.

Moreover, the ROC curve analysis showed that the proposed theta variable achieved high specificity while maintaining relatively high sensitivity for identifying the pathological status of patients, suggesting its potential utility as a diagnostic support tool.

This analysis enables the identification of patients who exhibit symptoms but do not meet the diagnostic cutoff criteria for pathology, which would allow them to receive treatment more appropriately tailored to their pathological group profile.

As shown in our results, individuals within the MP-S group appear to preserve an overall active cardiac vagal baroreflex function, as well as an active parasympathetic function, this suggests a limited degree of neuroinflammation (78); thus suggesting that this subgroup may have the greatest likelihood of responding favorably to therapeutic interventions aimed at reversing the neuroinflammation, therefore causing a decrease in symptoms and physiological effects of the disease. This evaluation will be a topic in a forthcoming publication.

Moreover, the MP Δ*θ* > 40 and MP *θ* < 40 groups exhibited attenuated baroreflex sensitivity (BRS) together with a reduced ellipse area. Notably, the MP *θ* < 40 group showed the greatest reduction in ellipse area, suggesting that this subgroup may represent the phenotype closest to baroreflex failure as seen by Keller et al. (51).

## 5 Limitations

The present exploratory study was performed with a limited sample size of patients diagnosed with Long-COVID who were admitted to our institution and is not fully representative of all COVID patients. A multicenter prospective study is required to properly validate our preliminary findings. Therefore, the present study should be considered hypothesis-generating. Nevertheless, the effect sizes and statistical differences were sufficiently large to support the main conclusions (e.g., low probability of the type I statistical error).

We are aware that beat-to-beat analysis is currently not standard in most clinics and our team is currently evaluating other ways to perform the proposed methodology through more accessible and elementary means.

## 6 Future directions

From this perspective, future studies should focus on validating the proposed BRS and *θ*-based subgroup classification in larger and more heterogeneous cohorts to evaluate the potential influence of age, sex, and initial disease severity and to confirm the reproducibility and generalizability of the identified thresholds and the bimodal distribution. In particular, longitudinal studies will be essential to determine whether these subgroups are associated with distinct recovery trajectories, symptom persistence, or differential responses to therapeutic interventions. Additionally, further research should evaluate the feasibility of implementing this graphical approach in routine clinical practice, including settings without specialized beat-to-beat monitoring equipment, as well as its potential integration into digital or web-based tools for broader clinical use.

Our group is undertaking the longitudinal evaluation of these parameters in a larger population with Long-COVID, which will allow further analyses and draw conclusions regarding the implementation of continuous blood pressure assessment in the initial approach to patients with Long-COVID symptoms.

Public health policies should be implemented to ensure this type of equipment is available in more clinics around the world, although this is still beyond the authors' reach. However, efforts are underway to expand the current methodology so it can be implemented through web-based tools.

## 7 Conclusions

This study evaluated orthostatic intolerance in a cohort of patients with Long-COVID using a new method that allows a more precise characterization based on BRS alteration. We identified a significant decrease in the BRS function in patients with Long-COVID, which was more pronounced during the orthostatic position and was accompanied by a higher heart rate and lower heart rate variability. This led us to suggest a reclassification of patient diagnoses into the MP-S, MP Δ*θ* > 40 and MP *θ* < 40 subgroups, where the greatest change was observed in the MP Δ*θ* > 40, corresponding to patients with the most severe symptoms.

The proposed classification supports the concept that BRS dysfunction in Long-COVID may evolve as a progressive deterioration that can be represented as a spectrum, closely associated with symptom burden and HRV alterations. These findings may provide a useful framework for the development of future diagnostic, prognostic, and therapeutic strategies.

Although this study is exploratory, applying our new methodology enabled us to understand the importance of BRS impairment and propose a novel classification approach that improves our understanding of autonomic dysfunction in patients with Long-COVID and other causes of dysautonomia.

## Data Availability

The datasets presented in this study can be found in online repositories. The names of the repository/repositories and accession number(s) can be found below: The datasets generated for this study can be found in the “Baroreflex Sensitivity Impairment In Long-COVID Patients” dataset, OSF, https://doi.org/10.17605/OSF.IO/HB6MN.
