# Supplementary material for: Baroreflex sensitivity impairment in Long-COVID patients: a diagnostic tool for classifying the autonomic dysfunction spectrum
Source: Front Cardiovasc Med. 2026 Jul 14;13:1830347. doi: 10.3389/fcvm.2026.1830347 (PMC13410891; doi:10.3389/fcvm.2026.1830347)
Supplement: Supplementary file 1 [file Supplementaryfile1.docx]

| **Name:** |  | | **Date:** |  |
| --- | --- | --- | --- | --- |
| **Date of birth:** |  | | **Weight:** |  |
| **Biological sex:** |  | | **Height:** |  |
| **Phone number:** |  | | **BMI:** |  |
| **E-mail:** |  | | **Age:** |  |
| **Medical record** | | | | |
| **Family history** | | | | |
| Paternal side: | | | | |
| Maternal side: | | | | |
| Siblings: | | | | |
| Children: | | | | |
| **Personal medical history** | | | | |
| Chronic diseases: | | | | |
| Medications: | | | | |
| **COVID history** | | | | |
| Number of times you have had COVID-19: | | Vaccinated before diagnosis: Yes / No | | |
| Date of diagnosis: | | Vaccine scheme used: | | |
| Method (of diagnosis): | | Adverse events: | | |
| Severity: | |  |  |  |
| Reported symptoms: | | | | |
| Treatment received: | | | | |

# Mark each of the following symptoms you have experienced after COVID-19:

| Constitutional symptoms | | |
| --- | --- | --- |
| Fatigue/tiredness | General malaise | Fever |
| Generalized pain | Weight gain | Weight loss |
| **Cardiovascular symptoms** | | |
| Palpitations | Chest pain | Dizziness when standing up |
| Neurological symptoms | | |
| Headache | Tinnitus (ringing) | Hearing difficulty |
| Numbness in hands | Numbness in feet | Seizures |
| Dizziness at rest | Thirst | “Brain fog” |
| Loss or change in smell | Loss or change in taste | Difficulty finding words |
| Difficulty concentrating | Difficulty remembering | Difficulty paying attention |
| Psychiatric symptoms | | |
| Nervousness | Insomnia | Loss of interest |
| Loss of appetite | Frequent forgetfulness | Panic attacks |
| Ophthalmologic symptoms | | |
| Blurred vision | Dry eyes | Difficulty focusing |
| Visual flashes/lights | Eye pain | Visual loss |
| Respiratory symptoms | | |
| Shortness of breath | Wheezing | Cough |
| Pain with deep breathing | Nasal congestion | Sore throat |
| **Musculoskeletal symptoms** | | |
| Muscle pain | Muscle weakness | Joint pain |
| Dermatological symptoms | | |
| Rashes/hives | Itching | Sun sensitivity |
| Dry skin | Paleness in hands or feet | Coloration changes |
| Changes in sweating | Easy bruising | Hair loss |
| Gastrointestinal symptoms | | |
| Irritable bowel | Diarrhea | Vomiting |
| Acid reflux | Postprandial bloating | Abdominal pain/cramps |
| Constipation | Nausea | Dry mouth |
| Genitourinary symptoms | | |
| Bladder spasms | Pain or burning with urination | Premenstrual pain |
| Difficulty starting urination | Difficulty maintaining urine stream | Irregular menstruation |
| Frequent urination | Erectile dysfunction | Decreased libido |

Others:

| **SS Score** | | | | | | | | | | | | |
| --- | --- | --- | --- | --- | --- | --- | --- | --- | --- | --- | --- | --- |
| Indicate the level of severity of the following symptoms during the **last week** using the following scale: | | | | | | | | | | | | |
|  | | | | | | Fatigue | | Non-restorative sleep | | | Cognitive difficulty | |
| None | | | | | | 0 | | 0 | | | 0 | |
| Mild/intermittent | | | | | | 1 | | 1 | | | 1 | |
| Considerate/frequent/moderate | | | | | | 2 | | 2 | | | 2 | |
| Severe/continuous/impactful | | | | | | 3 | | 3 | | | 3 | |
| **Total:** | | | | | |  | | | | | | |
| **COMPASS-31** | | |  | | | | | | | | | |
|  |  |  | | **Blood Pressure Measurement** | | | | |  | | | |
|  | **Day 1** |  | | **Day 2** | | | | | **Consultation Day** | | | |
| Morning | Afternoon | Night | | Morning | Afternoon | | Night | | Lying down | Standing | | Sitting |
|  |  |  | |  |  | |  | |  |  | |  |
| **Analysis and Commentary** | | | | | | | | | | | | |
|  | | | | | | | | | | | | |

***Score system:***

| Constitutional symptoms | | |
| --- | --- | --- |
| Fatigue/tiredness | General malaise | Fever |
| Generalized pain | Weight gain | Weight loss |
| **Cardiovascular symptoms** | | |
| Palpitations | Chest pain | Dizziness when standing up |
| Neurological symptoms | | |
| Headache | Tinnitus (ringing) | Hearing difficulty |
| Numbness in hands | Numbness in feet | Seizures |
| Dizziness at rest | Thirst | “Brain fog” |
| Loss or change in smell | Loss or change in taste | Difficulty finding words |
| Difficulty concentrating | Difficulty remembering | Difficulty paying attention |
| Psychiatric symptoms | | |
| Nervousness | Insomnia | Loss of interest |
| Loss of appetite | Frequent forgetfulness | Panic attacks |
| Ophthalmologic symptoms | | |
| Blurred vision | Dry eyes | Difficulty focusing |
| Visual flashes/lights | Eye pain | Visual loss |
| Respiratory symptoms | | |
| Shortness of breath | Wheezing | Cough |
| Pain with deep breathing | Nasal congestion | Sore throat |
| **Musculoskeletal symptoms** | | |
| Muscle pain | Muscle weakness | Joint pain |
| Dermatological symptoms | | |
| Rashes/hives | Itching | Sun sensitivity |
| Dry skin | Paleness in hands or feet | Coloration changes |
| Changes in sweating | Easy bruising | Hair loss |
| Gastrointestinal symptoms | | |
| Irritable bowel | Diarrhea | Vomiting |
| Acid reflux | Postprandial bloating | Abdominal pain/cramps |
| Constipation | Nausea | Dry mouth |
| Genitourinary symptoms | | |
| Bladder spasms | Pain or burning with urination | Premenstrual pain |
| Difficulty starting urination | Difficulty maintaining urine stream | Irregular menstruation |
| Frequent urination | Erectile dysfunction | Decreased libido |

| *Points* |  |
| --- | --- |
| *4 points* |  |
| *3 points* |  |
| *2 points* |  |
| *1 point* |  |
