# Supplementary material for: Baroreflex sensitivity impairment in Long-COVID patients: a diagnostic tool for classifying the autonomic dysfunction spectrum
Source: Front Cardiovasc Med. 2026 Jul 14;13:1830347. doi: 10.3389/fcvm.2026.1830347 (PMC13410891; doi:10.3389/fcvm.2026.1830347)
Supplement: Supplementary file 2 [file Supplementaryfile2.docx]

1. **Supplementary Methods 1:**
   1. **Study inclusion criteria:**

Patients presented symptoms of Long Covid, patients previously presented a positive Covid test (swab test and PCR), patient scored >30 in the Compass31+ questionnaire.

- 1. **Study exclusion criteria:**

Patients who had an acute Covid-19 infection during the selection process, patients who presented an autoimmune disorder, patients who were hospitalized at the time, and patients who were unable to stand up by themselves.

1. **Supplementary Methods 2:**
   1. **Alternate method for calculating the Theta angle:**

Following the adjustment, we normalized the data of the ΔSBP and ΔIBI by dividing each value by 10 and 100, respectively. Once this was done, we calculated the semi-major axis of the ellipse and obtain the rotation angle with respect to the horizontal axis by calculating the inverse tangent of the slope formed by the previously mentioned semi-axis to obtain what we refer to as the patient’s “Theta Angle”[º] (Fig. 1). A similar method was also developed in which the original delta values are not modified and the same angle is achieved.
