# Supplementary material for: Baroreflex sensitivity impairment in Long-COVID patients: a diagnostic tool for classifying the autonomic dysfunction spectrum
Source: Front Cardiovasc Med. 2026 Jul 14;13:1830347. doi: 10.3389/fcvm.2026.1830347 (PMC13410891; doi:10.3389/fcvm.2026.1830347)
Supplement: Supplementary file 3 [file Supplementaryfile3.docx]

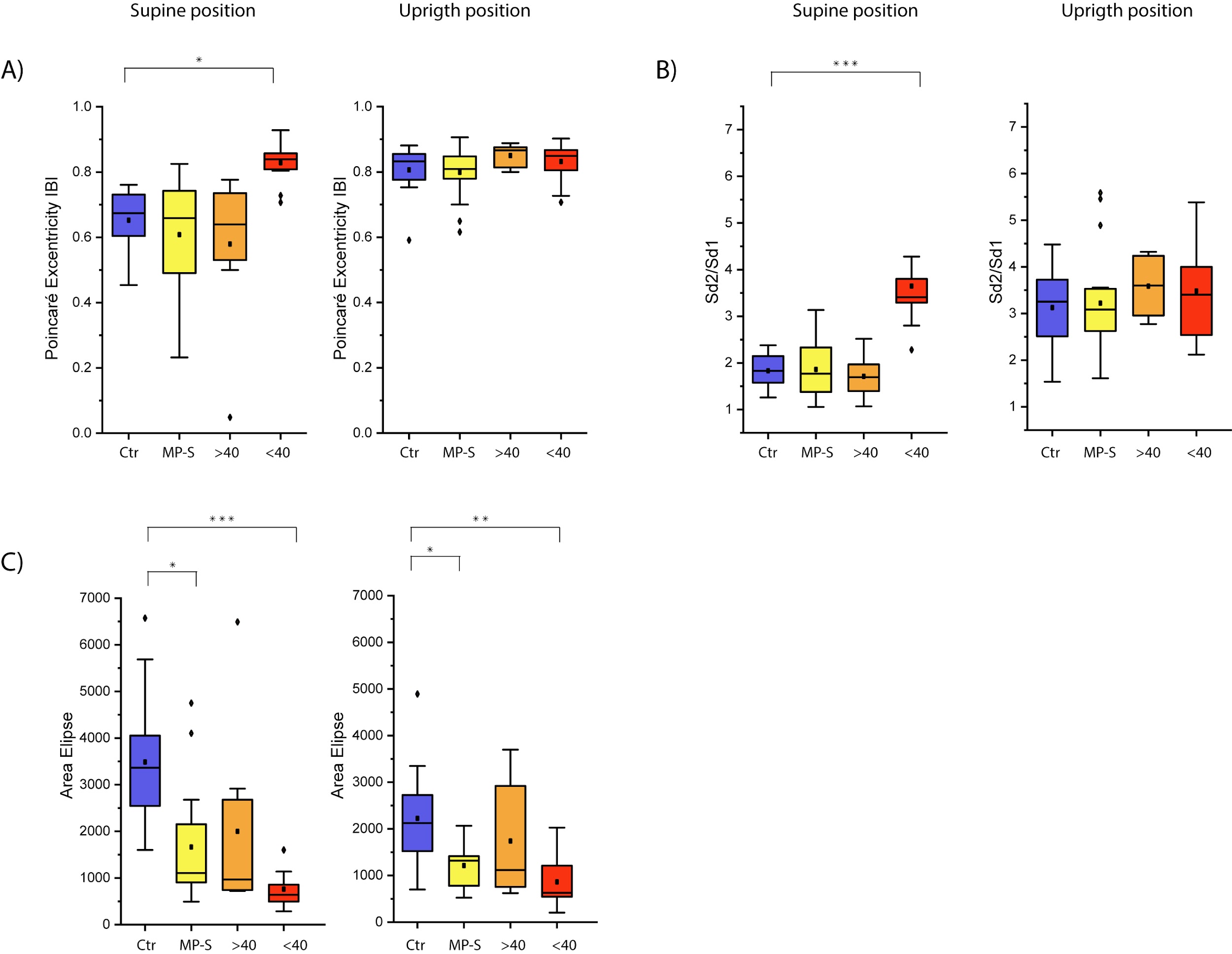


**Figure S1 Box plot of Poincaré and Theta Ellipse area for all groups**. Box plots with the data from Poincaré plots of each patient, such as eccentricity **(A)** and Sd2/Sd1 **(B)**; additionally, the plot for the Theta ellipse area **(C)** is shown. To indicate statistical significance with respect to the control group, an asterisk [*] was placed for a p<0.05, two asterisks [**] for p<0.001 and three asterisks [***] for p<0.0001
