# Supplementary material for: Baroreflex sensitivity impairment in Long-COVID patients: a diagnostic tool for classifying the autonomic dysfunction spectrum
Source: Front Cardiovasc Med. 2026 Jul 14;13:1830347. doi: 10.3389/fcvm.2026.1830347 (PMC13410891; doi:10.3389/fcvm.2026.1830347)
Supplement: Supplementary file 5 [file Supplementaryfile5.docx]

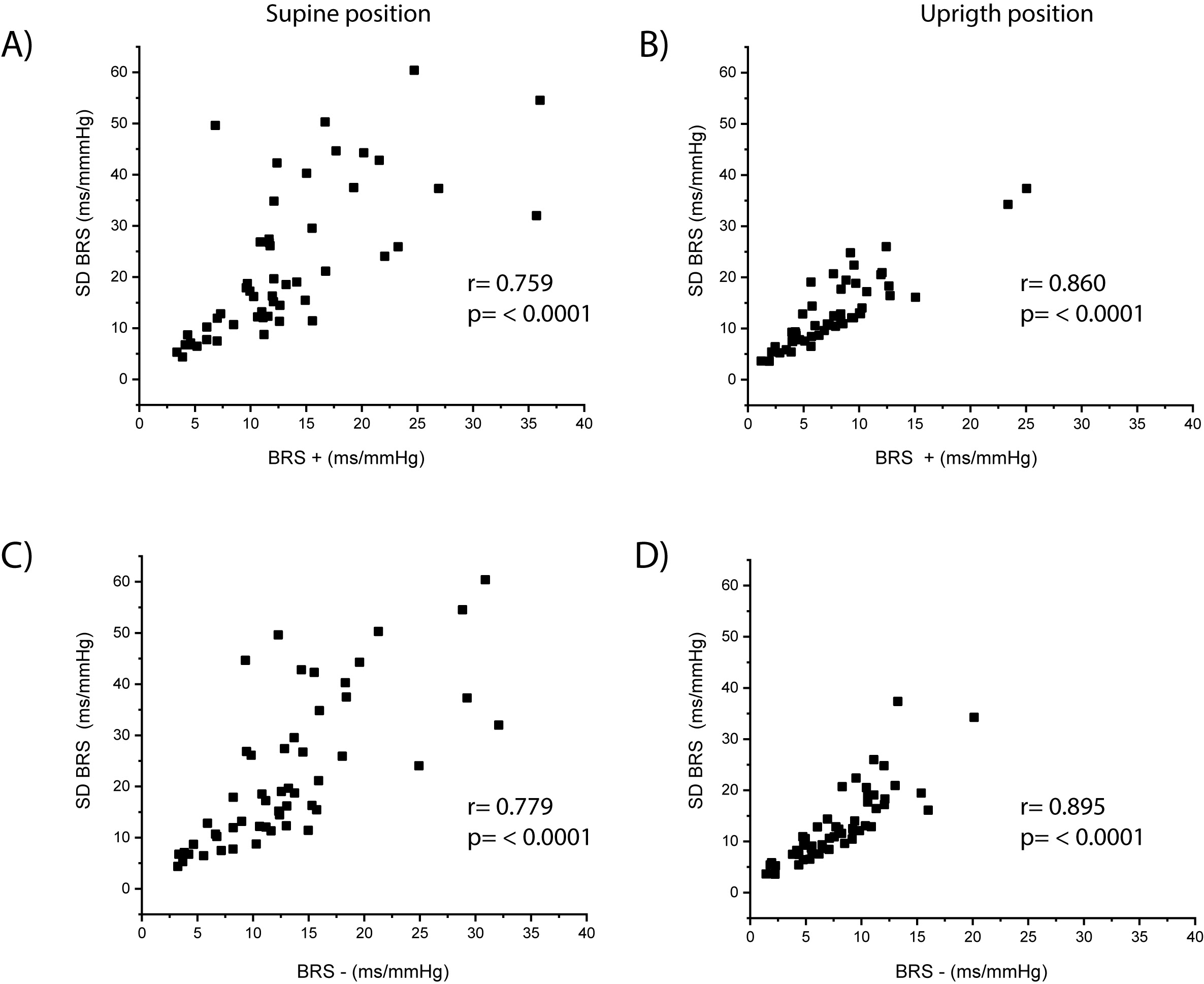


**Figure S3** **Relationship between the Sequence method and the SD BRS**. The values of the positive and negative sequences have been placed in the X-axis to compare them with the values of the proposed SD BRS, which have been placed over the Y-axis. Additionally, the Spearman’s R value was placed for each corresponding graph along with its p value
