# Supplementary material for: Baroreflex sensitivity impairment in Long-COVID patients: a diagnostic tool for classifying the autonomic dysfunction spectrum
Source: Front Cardiovasc Med. 2026 Jul 14;13:1830347. doi: 10.3389/fcvm.2026.1830347 (PMC13410891; doi:10.3389/fcvm.2026.1830347)
Supplement: Supplementary file 6 [file Supplementaryfile6.docx]

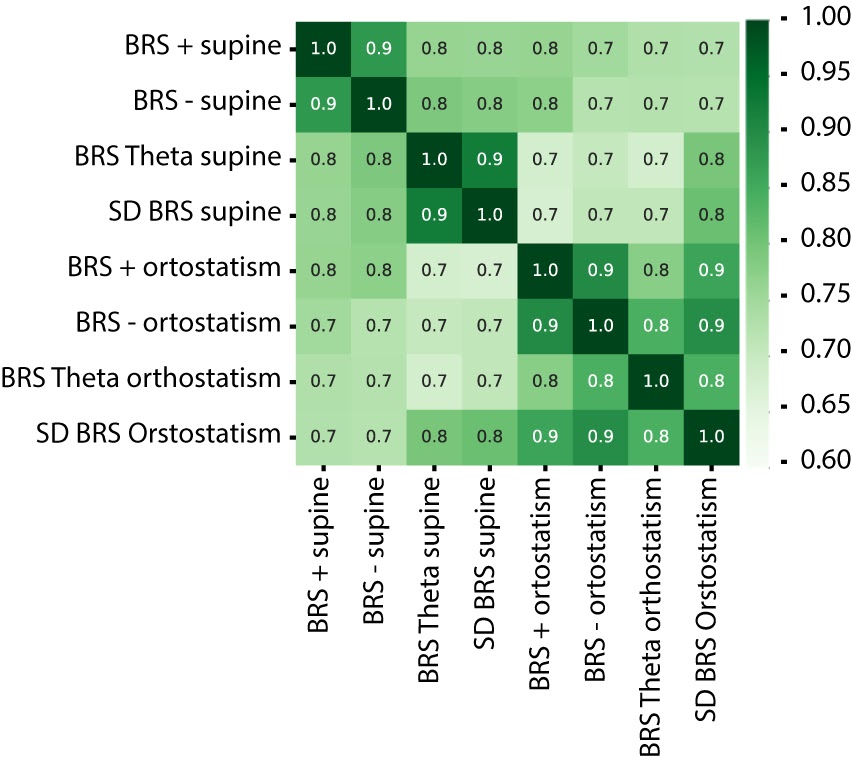


**Figure S4 Spearman matrix for the sequence method and proposed variables.** Matrix with the positive and negative sequences of the BRS along with the proposed Theta and SD BRS values during supine and upright positions, where a heatmap was applied to match the correlation values obtained using the Spearman’s test.
