# Supplementary material for: Baroreflex sensitivity impairment in Long-COVID patients: a diagnostic tool for classifying the autonomic dysfunction spectrum
Source: Front Cardiovasc Med. 2026 Jul 14;13:1830347. doi: 10.3389/fcvm.2026.1830347 (PMC13410891; doi:10.3389/fcvm.2026.1830347)
Supplement: Supplementary file 7 [file Supplementaryfile7.docx]

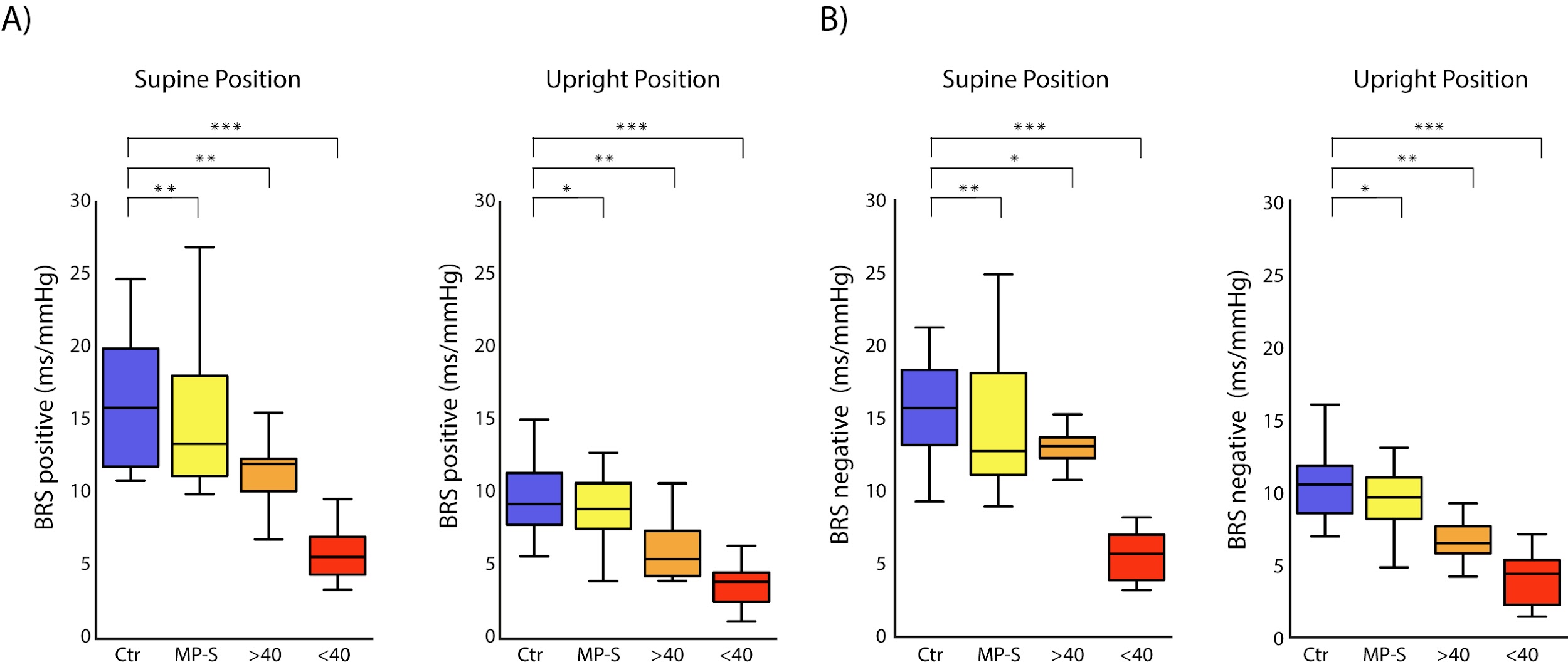


**Figure S5 Validation of BRS groups using the sequence method.** Box plot comparing the results of the BRS measurement performed with the sequence method and the proposed method for the selected groups; both methods yielding similar results. To indicate statistical significance to the control group, an asterisk [*] was placed for a p<0.05, two asterisks [**] for p<0.001 and three asterisks [***] for p<0.0001
