# Supplementary material for: Baroreflex sensitivity impairment in Long-COVID patients: a diagnostic tool for classifying the autonomic dysfunction spectrum
Source: Front Cardiovasc Med. 2026 Jul 14;13:1830347. doi: 10.3389/fcvm.2026.1830347 (PMC13410891; doi:10.3389/fcvm.2026.1830347)
Supplement: Supplementary file 8 [file Supplementaryfile8.docx]

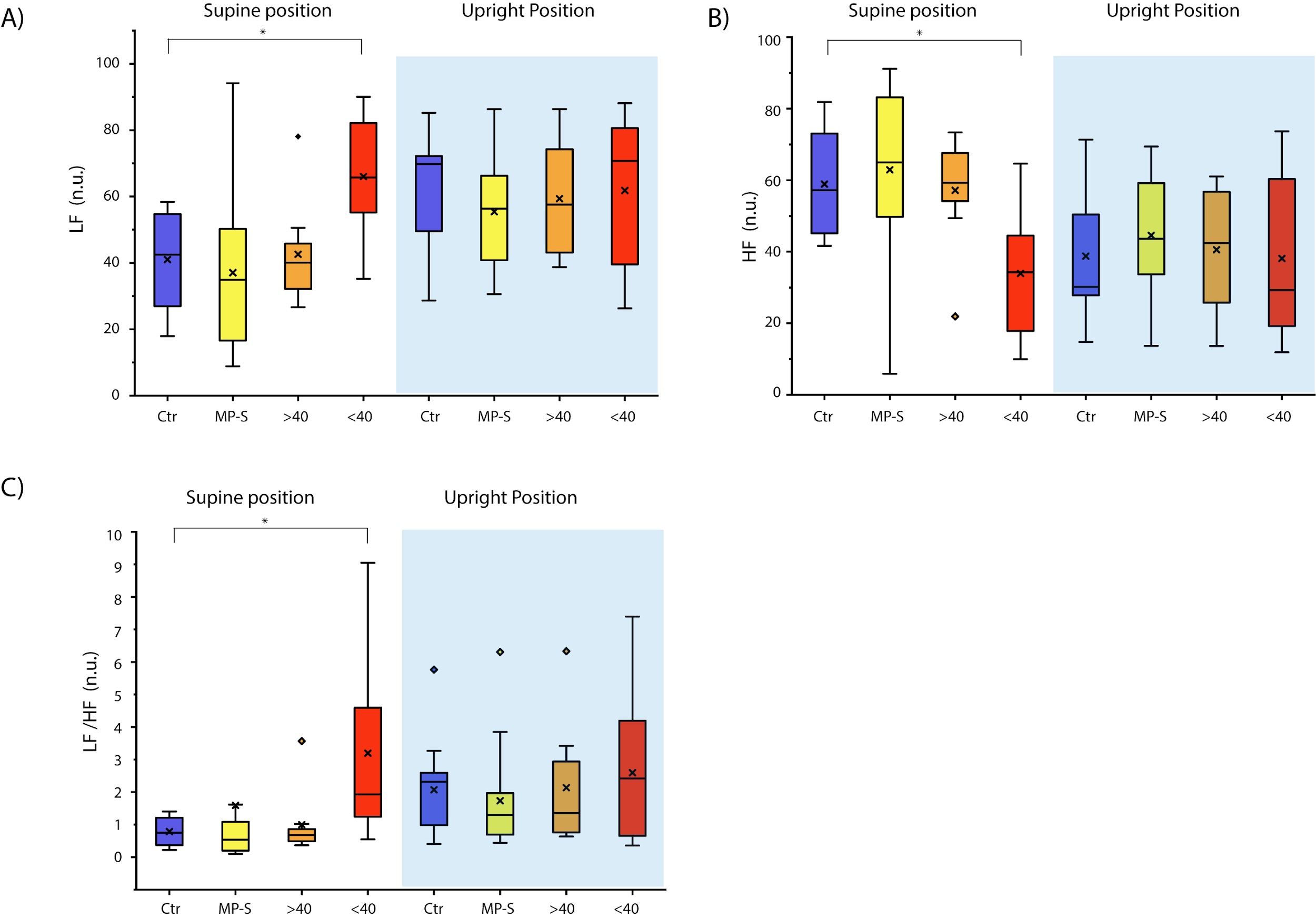


**Figure S6 Frequency domain variables comparison among groups.** Box plots for the measurements of LF n.u. **(A)**, HF n.u. **(B)** and LF/HF **(C)** ratio for all study groups during the supine and upright positions using the Mann Whitney test. During the supine position of the LF band, the MP θ<40 group is the only one that is significantly different from the control group; this is also true for the HF band and the LF/HF ratio. To indicate statistical significance with respect to the control group, an asterisk [*] was placed for a p<0.05.
